# Analysis of small RNA populations generated in peanut leaves after exogenous application of dsRNA and dsDNA targeting aflatoxin synthesis genes

**DOI:** 10.1038/s41598-020-70618-6

**Published:** 2020-08-14

**Authors:** Imana L. Power, Paola C. Faustinelli, Valerie A. Orner, Victor S. Sobolev, Renee S. Arias

**Affiliations:** 1 United States Department of Agriculture, Agricultural Research Service, National Peanut Research Laboratory (NPRL), Dawson, GA USA; 2 Present Address: Center for Agricultural Research in Suriname (CELOS), Paramaribo, Suriname

**Keywords:** Molecular engineering in plants, RNAi

## Abstract

Previously, we have shown that RNA interference (RNAi) can prevent aflatoxin accumulation in transformed peanuts. To explore aflatoxin control by exogenous delivery of double-strand RNA (dsRNA) it is necessary to understand the generation of small RNA (sRNA) populations. We sequenced 12 duplicate sRNA libraries of *in-vitro*-grown peanut plants, 24 and 48 h after exogenous application of five gene fragments (RNAi-5x) related to aflatoxin biosynthesis in *Aspergillus flavus*. RNAi-5x was applied either as double-stranded RNA (dsRNA) or RNAi plasmid DNA (dsDNA). Small interfering RNAs (siRNAs) derived from RNAi-5x were significantly more abundant at 48 h than at 24 h, and the majority mapped to the fragment of aflatoxin efflux-pump gene. RNAi-5x-specific siRNAs were significantly, three to fivefold, more abundant in dsDNA than dsRNA treatments. Further examination of known micro RNAs related to disease-resistance, showed significant down-regulation of miR399 and up-regulation of miR482 in leaves treated with dsDNA compared to the control. These results show that sRNA sequencing is useful to compare exogenous RNAi delivery methods on peanut plants, and to analyze the efficacy of molecular constructs to generate siRNAs against specific gene targets. This work lays the foundation for non-transgenic delivery of RNAi in controlling aflatoxins in peanut.

## Introduction

One of the major constraints in peanut production is aflatoxin contamination, resulting in yearly losses that can add up to millions of dollars^1^. Aflatoxins produced by *Aspergillus flavus* Link and *A. parasiticus* Speare are detrimental to human health, as they can lead to stunting in children^2^, immunosuppression, and acute hepatotoxicity^3^. *Aspergillus flavus* and *A. parasiticus* can infect and produce aflatoxins in other important agricultural crops as well^4^. Germplasm improvement, biological control, and crop management practices have so far been the main management strategies in peanut to combat aflatoxin contamination^5–7^ though the problem still persists. There are several reports of the successful implementation of gene silencing or RNA interference (RNAi) against plant pathogenic fungi^8–12^; Cooper and Campbell^8^ found lower pustule development in bean (*Phaseolus vulgaris*) plants that expressed RNA targeting rust (*Uromyces appendiculatus*) effector genes; and Ghag et al.^9^ developed transgenic banana (*Musa* spp.) lines expressing essential genes of *Fusarium oxysporum* f. sp. *cubense* and these transgenic lines were still disease-free eight months after inoculation. Options to reduce aflatoxin production using RNAi in crops have had varying levels of success^13–16^. Control of plant diseases through RNAi is aimed at degradation of target-specific pathogenic mRNA, resulting in silencing of that gene in the pathogen.

RNA interference (RNAi) is a biological process in which a double-stranded RNA (dsRNA) signal leads to silencing of a target gene^17^. The process involves several enzymes and starts with the recognition of a dsRNA signal that binds to a protein complex called DICER (DCL); this protein complex hydrolyzes the dsRNA into small RNAs (sRNAs). One strand of the sRNA is then loaded onto an Argonaute protein (AGO) to form the RNA-Induced-Silencing Complex (RISC), which cleaves and degrades mRNA that is complementary to the sRNA, resulting in silencing of the gene encoding the mRNA^17–20^. Small interfering RNAs (siRNAs) and micro RNAs (miRNAs) are the two main classes of sRNAs. Small interfering RNAs are derived from dsRNA and cleaved into 20–24 nucleotides by DCL2, DCL3 or DCL4, and miRNAs are derived from single-stranded RNA (ssRNA) and cleaved by DCL1 into 19–24 nucleotides^18–20^.

Reduction of aflatoxin accumulation in peanut using RNAi is one of our focus areas of research, and as part of that effort, peanut plants have been transformed to generate dsRNA that simultaneously targets five genes in the aflatoxin biosynthesis pathway of *A. flavus*^13,15^. Several transformed peanut lines were generated that reduced the accumulation of aflatoxin by 74% to 100% compared to non-transformed control peanut plants^13,15^. Since there was a range in ability to reduce the aflatoxin accumulation in the transformed peanut lines, we previously performed high-throughput sequencing of sRNAs to identify transgene-derived sRNAs, and possible differences in abundance of sRNA populations in the control and transformed peanut lines^15^. However, very few sRNAs specific to the transgene and to the genome of *A. flavus* were identified, and they were in low abundance^15^. These results had raised several questions, e.g*.*: were construct-specific sRNAs generated? If so, what was the timeframe for their production, and for how long were these sRNAs detectable? In addition, it was important to know whether sRNA were generated by all five gene fragments in the RNAi construct or only some of them.

The aim of the current study was to begin answering some of the listed questions within a very short timeframe, 24–48 h after delivery of RNAi-trigger signals. First we examined the efficiency of two methods of introduction of RNAi signals for silencing aflatoxin biosynthesis genes into peanut plants, by measuring the production of construct-specific sRNAs in plant tissue. Here we used high-throughput sequencing of sRNAs after exogenous application of two types of RNAi constructs in peanut tissue, and analyzed siRNA generation at the site of application after 24 and 48 h.

## Methods

### Plant material and nucleic acid preparation

**Figure 1:**
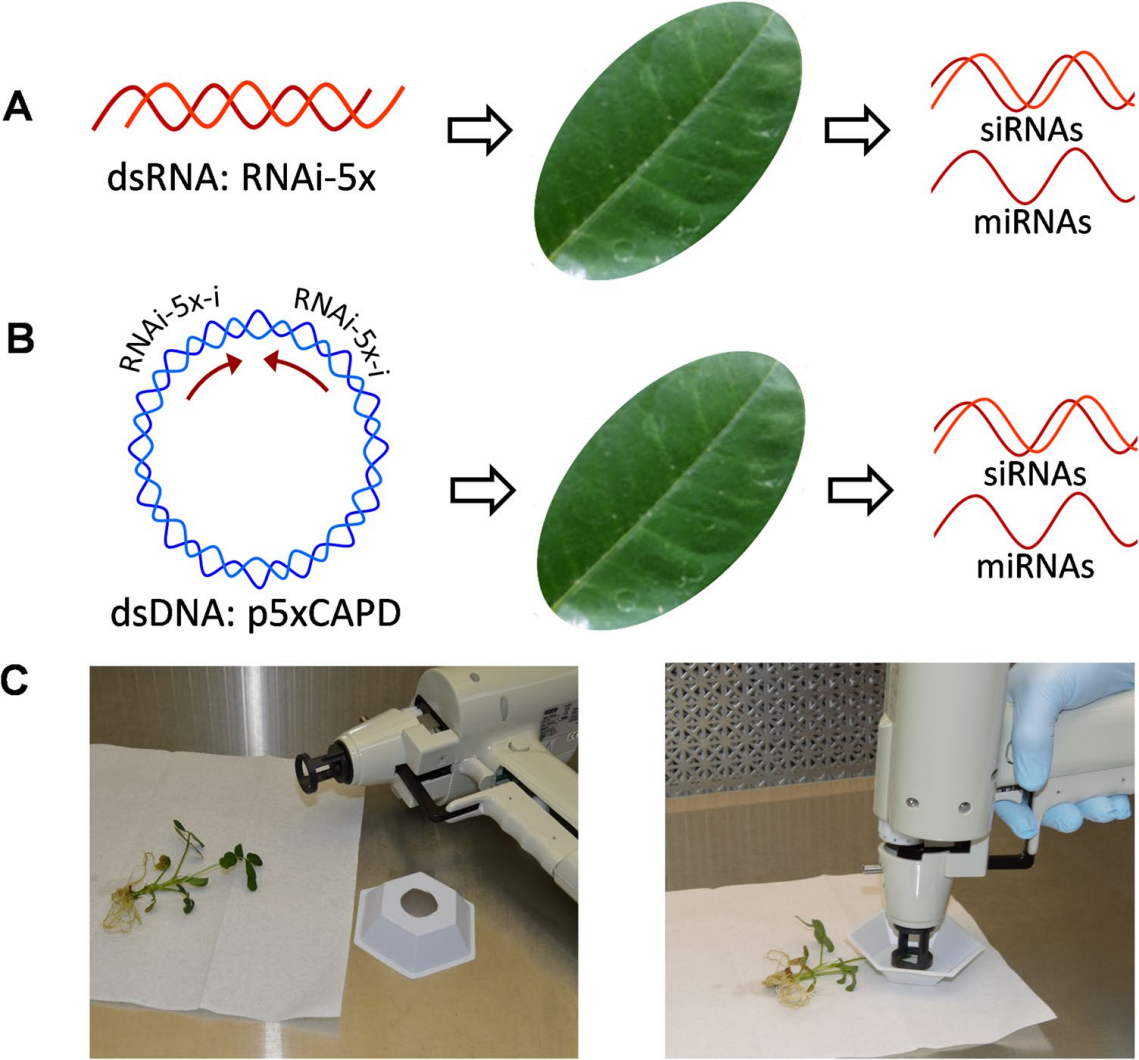
Schematic representation of the treatments using particle bombardment with the dsRNA insert. (**A**) Double-stranded RNA (dsRNA) of RNAi-5x insert. (**B**) Plasmid DNA (p5xCAPD) containing inverted repeats to generate dsDNA. (**C**) *In-vitro*-grown peanut plants, carved weighing dish, and Helios gene gun used for delivery of RNAi-5x insert.

Peanut seeds of the variety Georgia-06G^21^ were germinated and grown on MSO medium consisting of Murashige and Skoog^22^, salts and vitamins, 3% sucrose, and 0.8% agar (Sigma-Aldrich, St. Louis, MO, USA); the plants were kept at 26 ± 2 °C, and a 16/8 h light/dark photoperiod. In this study, the first fully expanded leaves of *in-vitro*-grown plants were treated with dsRNA or plasmid DNA containing an RNAi fragment. The plants were removed from the medium under aseptic conditions before the application of nucleic acids, and rapidly returned to fresh growth medium after treatment (Fig. 1).

Treatments consisted of delivering an RNAi fragment, either as a complete plasmid construct (p5xCAPD, dsDNA), or as a double-stranded RNA (RNAi-5x, dsRNA). Plasmid p5xCAPD (dsDNA) was obtained by first integrating small fragments (~ 78 bp ea.) of five genes from *A. flavus* involved in the biosynthesis of aflatoxins, to make the RNAi-5x insert (393 bp) (Table 1). RNAi-5x insert was initially cloned in vector TOPO4 (Invitrogen, Carlsbad, CA, USA) and named p5x-TOPO4^13^. Then, the RNAi-5x insert was subcloned into pENTR1A (Invitrogen, Carlsbad, CA, USA) and transferred as inverted repeats flanking the potato PIV2 intron into plasmid pCAPD (NCBI accession: KC176455.1)^23^ using LR-clonase (Invitrogen, Carlsbad, CA, USA) to form dsDNA^13^. The five targeted genes in RNAi-5x  insert were AFL2G_07223 (*afl*S or *afl*J), AFL2G_07224 (*afl*R), AFL2G_07228 (*afl*C/*pks*A/*pks*L1), AFL2G_07731 (*pes*1), and AFL2G_05027 (aflatoxin efflux pump, *afl*ep): the names correspond to accessions in the genome annotation of *A. flavus* (BROAD Institute, Cambridge, MA), other names are in parentheses^24^. These target genes are involved in mycotoxin synthesis of *Aspergillus flavus*, in particular in the production of aflatoxin. *afl*R and *afl*S or *afl*J are regulators of aflatoxin biosynthesis proteins in the aflatoxin cluster, and *afl*C/*pks*A/*pks*L1 is a polyketide synthase that synthesizes norsolorinic acid, an aflatoxin precursor and first step in aflatoxin synthesis. The aflatoxin efflux pump is important in moving aflatoxin out of the cells of *A. flavus*, and *pes*1 is a non-ribosomal peptide synthase involved in fungal virulence, and hypothetically could be involved in the synthesis of cyclopiazonic acid, a neurotoxin^24^. For a representation of the gene fragments in the dsDNA construct, see Arias et al.^13^

**Table 1:** Sequences of the gene fragments used in the dsRNA and ds DNA constructs.

Gene	Length	Sequence of the gene fragment
AFL2G_07224 (*afl*R)	84	GCCAGCTCAAAAGTGCGATGCACCAAGGAGAAACCGGCCTGTGCTCGGTGTATCGAACGTGGTCTTGCCTGTCAATACATGGTC
AFL2G_05027 (aflatoxin efflux pump)	76	GTATTTGTGACCATGTTTCTGGTGGCATTGGACCGTCTTGTCATCTCTACAGCCATTCCCCAGATCACGGACGAAT
AFL2G_07223 (*afl*S or *afl*J)	72	CCCCTGCATCTACGCGCACGCATCACTTGGGGTACCCGTCTATCAACAGCAACACAACCTGTCCTCGATGCG
AFL2G_07228 (*afl*C/*pks*A/*pks*L1)	80	GCTCAATCAAGGCAACTCTTTCTCTTCGGCGATCAGACAGCGGATTTTGTTCCCAAGCTCCGCAGTTTACTATCCGTCCA
AFL2G_07731 (*pes*1)	81	GGCCACGGTAGGGAGGTTTGGGACTCTGGAATTGACATTTCGCGTACTGTTGGGTGGTTTACCATTCTGCACCCAATAACC

The dsRNA (RNAi-5x) was synthesized by *in-vitro* transcription using two different plasmids coding for the RNAi-5x insert in opposite directions. The 393-bp DNA containing fragments of the five aflatoxin-related genes was PCR amplified using as template p5x-TOPO4^13^ and primers DirAll-cacc-Fw: 5′-CACCGCCAGCTCAAAAGTGCGATGC-3′ (stock #34) and DirAll- Nco-Rv: 5′-ATGCCATGGGGTTATTGGGTGCAGAATGG-3′ (stock #33). For the opposite orientation, the fragment was amplified using primers DirAll-inv-caccFw: 5′-CACCGGTTATTGGGTGCAGAATGG-3′ (stock #370), and primer Dir-1: 5′-GCCAGCTCAAAAGTGCGATGCACCAAGGAGAAACCGGCCTGTGCTCGGTGTATCGAACGTGGTCTTGCCTGTCAATACATGGTCGTATTTGTGACCATGTTTCTGGTGGCATTGGACCGTCTTGTCATCTCTACAGCCATTCCCCAGATCACGGACGAATCCCCTGCATCTACGCGCACGCATCACTTGGGGTACCCGT-3′ (stock #37) to directionally clone the fragments into pET161 vector (Invitrogen, Carlsbad, CA, USA) under the control of the T7 promoter. PCR amplification was performed in 20 µl total volume, containing 2 µl of 10 × High Fidelity PCR buffer (Thermo Fisher Scientific Inc., Waltham, MA, USA), 0.4 µl of 10 mM dNTPs, 0.8 µl of 50 mM MgSO_4_, 0.08 µl Platinum *Taq* DNA polymerase High Fidelity (5U/µl) (Thermo Fisher Scientific Inc., Waltham, MA, USA), 0.2 µl of each forward and reverse primer (10 µM), 5.0 µl DNA template, and 11.32 µl RNase-DNase free water. Amplification conditions were: initial denaturing step at 98 °C for 30 s followed by 35 cycles of 98 °C for 5 s, 63 °C for 5 s and 72 °C for 15 s, and a final extension at 72 °C for 5 min. Purified PCR products (QIAquick PCR Purification kit; Qiagen, Germantown, MD, USA) were ligated into pET161 plasmid, using the Champion pET161 Directional TOPO Expression kit (Thermo Fisher Scientific Inc., Waltham, MA, USA), and transformed into TOP10 competent cells (Thermo Fisher Scientific Inc., USA). Candidate clones were confirmed by sequencing, using forward and reverse universal T7 primers for pET161. Two plasmids, each containing two opposite orientations of RNAi-5x, were linearized with restriction enzyme *Hind*III (New England Biolabs Inc., Ipswich, MA, USA), and 1 µg of each linearized plasmid was transcribed using Ampliscribe T7-*Flash* Transcription Kit (Epicentre Biotechnologies, Madison, WI, USA) followed by treatment with DNase. Products of restriction digestion and ligation were sodium-acetate-isopropanol precipitated before each step. To obtain the dsRNA construct, equal molar concentrations of transcribed RNA strands of opposite orientations were mixed together, incubated at 100 °C for 1 min, and cooled at room temperature for 5 min. Quality and quantity of the dsRNA were examined on a 1% agarose gel and a NanoDrop spectrophotometer (Thermo Fisher Scientific Inc., Waltham, MA, USA).

### Cartridge preparation and delivery of nucleic acids

The two nucleic acids (dsDNA and dsRNA) were separately coated onto 0.6 µm ∅ gold microcarriers (Bio-Rad Laboratories Inc., Irvine, CA, USA), according to the Helios Gene Gun System Instruction Manual (Bio-Rad Laboratories Inc., Irvine, CA, USA), using 1 M CaCl_2_, 0.05 mg/ml polyvinylpyrrolidone (PVP: 360,000 MW; Sigma-Aldrich, St. Louis, MO, USA) in 100% ethanol, and 0.05 M spermidine (Sigma-Aldrich, St. Louis, MO, USA). Briefly, 373.5 µg nucleic acid in 100 µl (plasmid or dsRNA) per 25 mg 0.6 µm gold microcarriers, 100 µl of 0.05 M spermidine (5 µmol) and 100 µl 1 M CaCl_2_ (100 µmol) were mixed together in 300 µl total volume, then, incubated at room temperature to associate the nucleic acid with gold particles. For control treatments, gold particles, 100 µl deionized water, and 100 µl 0.05 M spermidine were used instead of nucleic acid. After centrifuging the suspension, the pellet was washed with 100% ethanol, and resuspended in 0.05 mg/ml PVP in 100% ethanol. The gold suspension was then drawn through a nitrogen-dried Gold-Coat tubing (Bio-Rad Laboratories Inc., Irvine, CA, USA) in the Tubing Prep Station (Bio-Rad Laboratories Inc., Irvine, CA, USA). After the gold particles were settled for 10 min, the ethanol was slowly removed from the tubing, and the particles were spread onto the inner surface of the tubing by continuous rotation of the tube. After rotating for about 30 s, the tube was dried with compressed nitrogen air at 0.3–0.4 L per minute, while rotating the tube for a total of 5 min. The coated tube was cut into 2.5 cm pieces (cartridges) using the tubing cutter (Bio-Rad Laboratories Inc., Irvine, CA, USA), and the cartridges were stored at − 80 °C until use. Each cartridge is estimated to contain 13.5 µg nucleic acid coated onto 0.5 mg gold microcarriers.

On the day of the experiment, *in-vitro*-grown peanut plants were placed in a laminar flow hood under sterile conditions (Fig. 1); each plant to be treated was removed from the agar medium, placed on a sterile paper towel, the leaflets were flattened and held in place using a sterile disposable polystyrene weighing dish (Sigma-Aldrich, St. Louis, MO, USA). Other leaves and leaflets were protected by covering the to-be-treated leaflet with another sterile weighing dish that contained a cutout of the circumference size of the barrel of the Helios Gene Gun, approximately 3 cm diameter. The cartridges were discharged on the abaxial area of the peanut leaflets. The treatments were (1) control: gold particles coated with deionized water instead of nucleic acid, (2) RNAi-5x: gold particles coated with dsRNA (RNAi-5x insert as double-stranded RNA), and (3) dsDNA: gold particles coated with the plasmid construct p5xCAPD. The helium pressure was set at 200 psi, and the nucleic acid loading ratio was 13.5 µg nucleic acid per shot (0.5 mg gold). Separate barrels and cartridge holders were used for each treatment to avoid any potential cross contamination. Each leaflet was shot only one time, and only one leaf per plant was used in the experiment. Plants were immediately returned to fresh medium after treatment, and incubated at 26 ± 2 °C on a 16/8 h light/dark photoperiod. Leaflets were treated either on day 1 or on day 2, and all the samples were collected on day 3. Thus, leaflets treated on day 1 were considered 48-h incubation, and those treated on day 2 were considered 24-h incubation.

### Sampling and RNA extraction

After 24 and 48 h of incubation, leaflets were sampled from the control and the two treatments, then frozen in liquid nitrogen, and stored at − 80 °C until RNA extraction within two days after sampling. Total RNA was extracted from these leaves (control, RNAi-5x, and dsDNA) using the Direct-zol RNA miniprep kit (Zymo Research, Irvine, CA, USA), according to manufacturer’s instructions. Leaves were ground in TRI Reagent (Zymo Research, Irvine, CA, USA) using a bead ruptor (OMNI International, Kennesaw, GA, USA), placed on ice and then continued with the Direct-zol procedure. Only one RNA sample was extracted from each plant, thus duplicate samples corresponded to two different plants that received the same treatment (control, dsRNA, and dsDNA) at 24 and 48 h post bombardment (hpb), totaling 12 RNA extractions from 12 plants.

### High throughput sequencing, annotation, and differential expression of small RNAs

High throughput sequencing, annotation, and differential expression of small RNAs was conducted as described previously^15^. High throughput sequencing of small RNA (sRNA) for each sample, totaling 12 libraries was performed at LC Sciences (Houston, TX), on the Illumina GAIIX platform. Unmappable reads [those containing adapter sequences or shorter than 15 nucleotides (nt)], and reads that mapped to mRNA (https://www.broadinstitute.org/annotation/genome/aspergillus group/MultiDownloads.html), Rfam (https://rfam.janelia.org) or Repbase (https://www.girinst.org/repbase), were not included in the analysis. The remaining mappable reads, 15–45 nt in length, were mapped to pre-miRNA and mature miRNAs in miRbase v21.0 (ftp://mirbase.org/pub/mirbase/CURRENT/), as well as to the genomes of the diploid parents of cultivated peanut, *Arachis hypogaea* L. (*A. duranensis* and *A. ipaënsis*), using Bowtie 2.0 (https://bowtie-bio.sourceforge.net/bowtie2/index.shtml), to identify known miRNAs. The mappable reads that did not map to miRBase, were mapped to the RNAi-5x insert to identify siRNAs possibly derived from the dsRNA insert. Normalized reads were used to determine the differential expression of sRNAs. The fold-change between treated and control leaves was calculated as: fold-change = Log_2_ (reads in treated/ reads in control). Small RNAs were considered upregulated if Log_2_ ≥ 2, and downregulated if Log_2_ ≤ 2, for p ≤ 0.01^15^.

### Validation of sRNAs by stemloop qRT-PCR

For validation of the sequencing results the experiment was repeated. Few small RNAs were tested using stem-loop primers and QuantiTect One-step real time PCR with SYBR Green Cat No. 204243 (Qiagen, Germantown, MD, USA). The stemloop primers were designed as described previously^25^. Stemloop, forward and reverse primers, and synthetic sequences of the small RNAs that were validated (the DNA oligonucleotides), were obtained from Integrated DNA Technologies (IDT, Coralville, Iowa, USA) (Table 2). To test the primers, the DNA oligonucleotide was used as template in tenfold dilutions from 66.67 ng to 0.67 pg in 25 µL reactions, consisting of 12.5 µL 2 X QuantiTect SYBR Green; 0.5 µM of each forward, reverse, and stemloop primer; 0.25 µL QuantiTect RT Mix; 10 µL DNA oligonucleotide template; nuclease-free water to adjust to 25 µL total volume reaction. PCR conditions were used according to manufacturer’s recommendations. This initial test was run in a QuantStudio 7 Flex Real-Time PCR and analyzed with QuantStudio Real-Time PCR Software v1.3 (QuantStudio 3 and 5 Real-Time PCR System Software | Thermo Fisher Scientific—US) (both from Applied Biosystems, Waltham, MA, USA).

**Table 2:** Primer sequences used for stemloop qRTPCR.

siRNA/reference miRNA	Forward primer	Stemloop primer
siRNA_186906	GCGGCGGGTGCCTGTCAATA	GTC GTA TCC AGT GCA GGG TCC GAG GTA TTC GCA CTG GAT ACG ACTACGAC
siRNA_141931	GCGGCGGGCTCGATGCGGCT	GTC GTA TCC AGT GCA GGG TCC GAG GTA TTC GCA CTG GAT ACG ACGCCTTG
siRNA_568285	GCGGCGGGATGCGGCTCAAT	GTC GTA TCC AGT GCA GGG TCC GAG GTA TTC GCA CTG GAT ACG ACAGAGTT
gma-miR166a-3p	GCGGCGGGTCGGACCAGGCT	GTC GTA TCC AGT GCA GGG TCC GAG GTA TTC GCA CTG GAT ACG ACGGGGAA
**Universal reverse primer**		CCA GTG CAG GGT CCG AGG TA

Following the same protocol, total RNA of two biological samples were analyzed in triplicate using 150 ng of RNA template diluted in 10 µL, using 25 µL reactions. The DNA oligonucleotide template used as positive control was at 6.6 pg per reaction, and nuclease-free water (without template) was used as negative control, both in triplicate. The same amplification conditions and software used for primer tests was used to analyze the samples.

## Results

### High throughput sequencing of small RNAs

**Figure 2:**
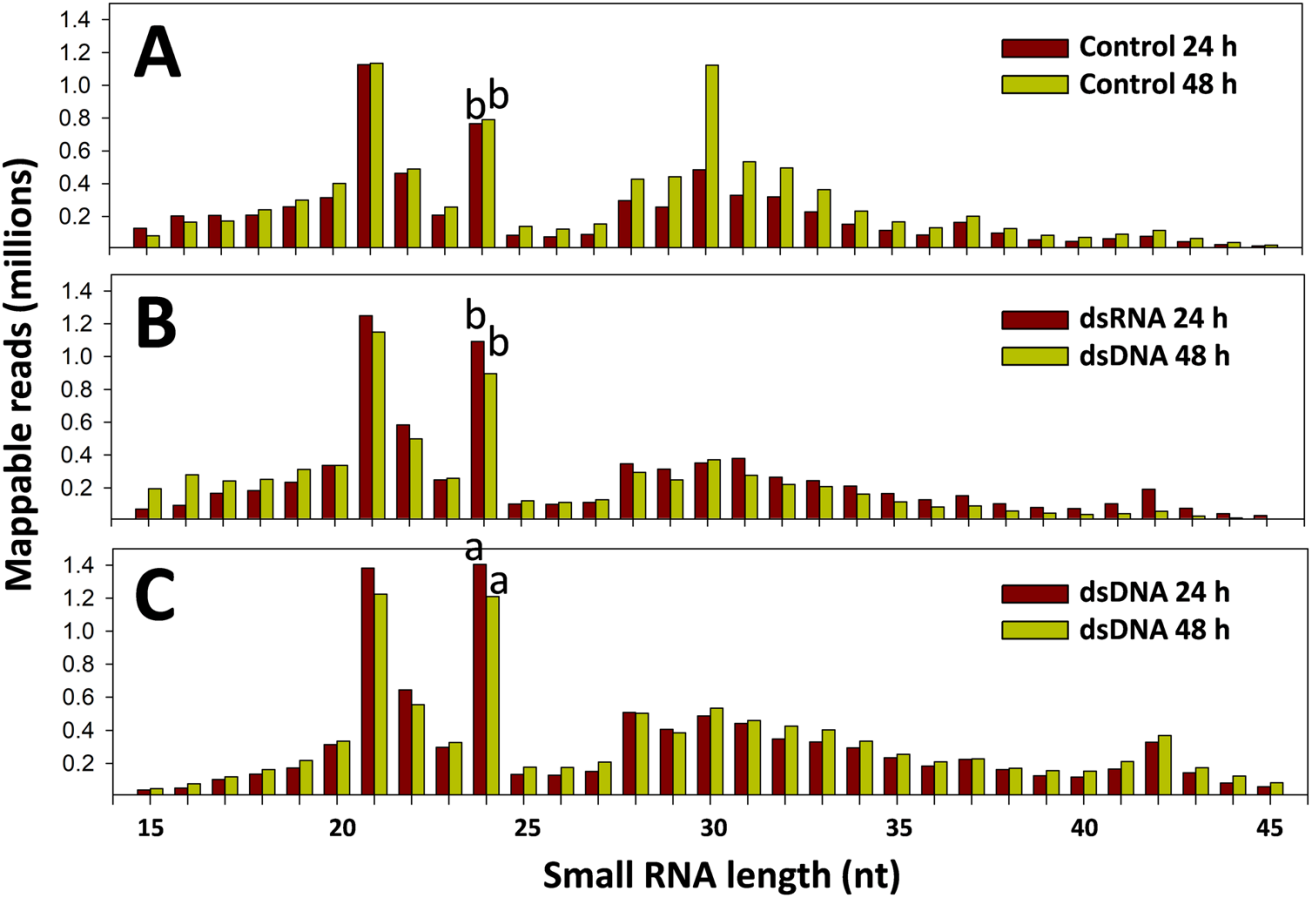
Length distribution (15–45 nt) of small RNAs in libraries of control, dsRNA, and dsDNA treatments, after 24 and 48 h incubation. (**A**) Control. (**B**) dsRNA. (**C**) dsDNA. Data represent the average of two libraries per treatment. Letters shown on bars at 24 nt for 24 and 48 h indicate the statistical comparison of mean values of normalized number of reads in control vs. treatments. Both at 24 and 48 h the number of 24 nt reads was significantly more abundant in control (p < 0.01) that in the treatments.

Twelve small RNA (sRNA) libraries were sequenced from peanut leaf samples of three treatments (control, dsRNA (RNA-5x), and dsDNA (p5xCAPD) treatments), at two incubation periods (24 and 48 h post bombardment), all in duplicate, and the data were normalized. After removal of adapters, removal of low-quality reads and retaining reads of 15–45 nucleotides (nt) in length, the obtained mappable reads ranged from 7 to 10 million reads for each of the libraries (Table 3). In all the libraries, the most frequent sRNA were 21 and 24 nt in length (Fig. 2A–C). In dsDNA the number of 24 nt sRNAs was significantly higher than in the control, both at 24 and 48 h, respectively (p ≤ 0.0078, p ≤ 0.0066, respectively) (Fig. 2A–C).

**Table 3:** Mappable reads (15–45 nucleotides) in 12 libraries: control, dsRNA and dsDNA treatments after 24 and 48 h incubation post bombardment (h), all in duplicate. Normalized read numbers are displayed.

	Abundance control 24 h	Abundance control 48 h	Abundance dsRNA 24 h	Abundance dsRNA 48 h	Abundance dsDNA 24 h	Abundance dsDNA 48 h
Rep^a^ 1	6.680	7.950	7.866	6.383	9.241	8.492
Rep 2	7.331	10.432	7.784	7.896	9.939	11.520

^a^Rep: replicate. Data after normalization and removal of adapters. Abundance in millions of reads.

### Analysis of known miRNAs

**Figure 3:**
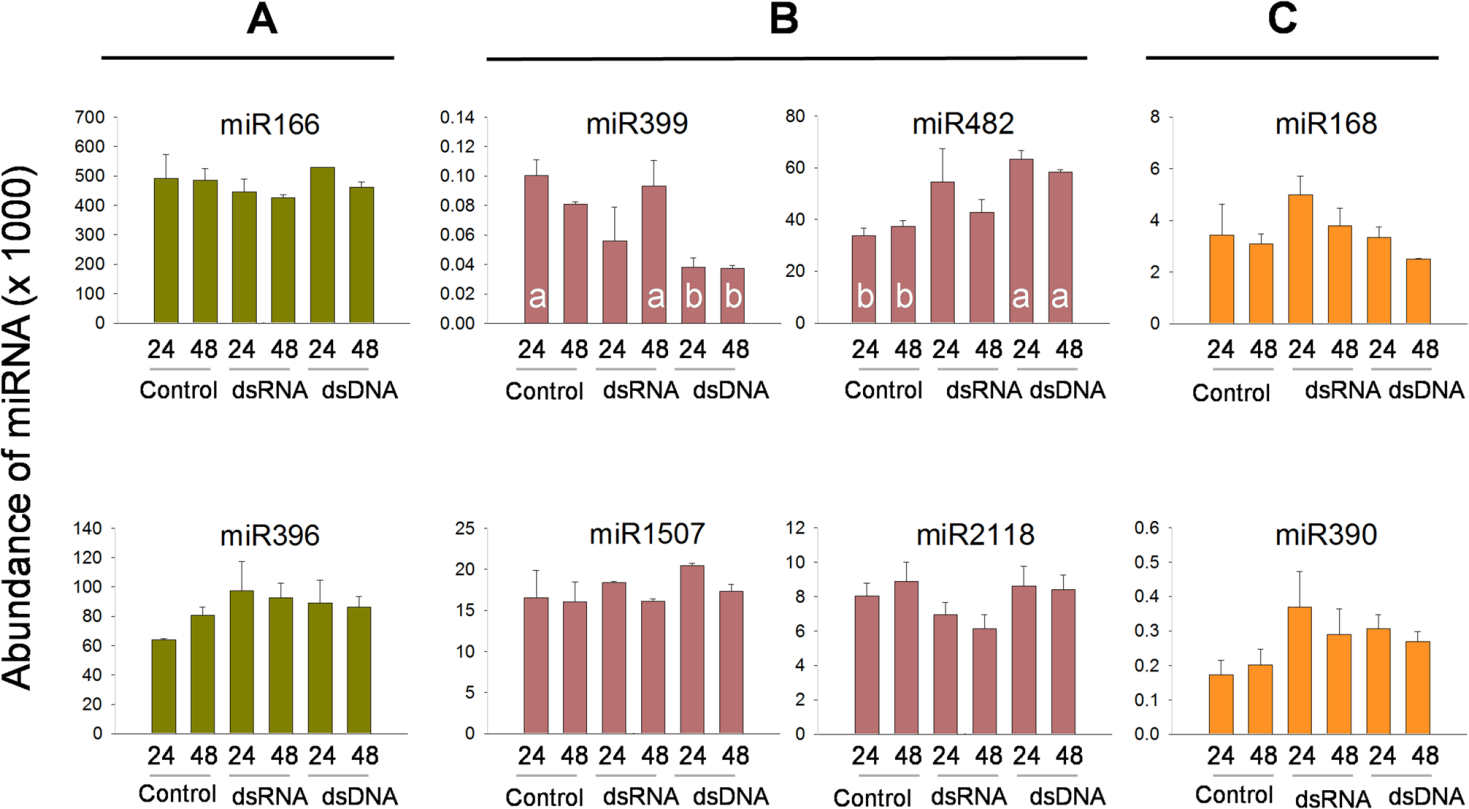
Abundance of selected known miRNAs. (**A**) miRNAs with the highest abundance. (**B**) miRNAs targeting disease resistance genes. (**C**) miRNAs targeting AGO1 and TAS3. Different letters within the histogram bars indicate statistically significant differences p ≤ 0.05.

The generated libraries were mapped to miRBase v21 to identify known peanut plant miRNAs possibly affected by the treatments. A total of 72 and 97 known miRNA families were identified at 24 and 48 h, respectively, in all libraries combined (control, dsRNA, and dsDNA treatments) (Supplementary Table S1). The majority of the miRNA families were represented by single family members: 38 miRNA families were represented by single family members at 24 h and 63 miRNA families were represented by single family members at 48 h; four miRNA families were represented by 10 or more members (Supplementary Table S1). The miR156 family contained the largest number of members (21 at 24 h and 22 at 48 h), followed by the miR166 family (15 members at both incubation periods), in all libraries (Supplementary Table S1). The expression levels of the miRNA families ranged from one or two reads, to more than 500,000 reads in the different libraries; the miR166 family had the highest abundance, followed by miR396, at both incubation periods, in all libraries (Supplementary Table S1, Fig. 3A). The miRNA families detected in this study already existed in the miRBase v21 database, or had been reported previously^15,26–30^. Seventeen of the 21 peanut miRNAs annotated in miRBase v21 were detected in the libraries analyzed in the present study (Supplementary Table S1).

To determine if the introduced RNAi constructs affected the expression of known miRNAs, the fold-change of miRNA expression in dsRNA and dsDNA libraries was compared to control treatments at p ≤ 0.01. In the libraries of the dsRNA treatment, some known miRNAs were differentially expressed, seven of them being mostly down-regulated, at 24 h, and six of them at 48 h, all up-regulated (Supplementary Table S2). In the libraries of the dsDNA treatments, 15 known miRNAs were differentially expressed, mostly down-regulated, at 24 h, and all nine differentially expressed at 48 h were down-regulated (Supplementary Table S2). Of the miRNA families miR399, miR482, miR1507, and miR2118, targeting disease resistance genes^27^, miR399 was significantly down-regulated in dsDNA treated leaves, and miR482 was up-regulated in this treatment (p ≤ 0.05) (Fig. 3B). The miRNA families involved in RNAi processes, miR168 targeting AGO1, and miR390 targeting TAS3^27^ did not show significant changes for any of the treatments and incubation (Fig. 3C).

More than 90% of the known miRNAs had uridine (U) as the 5′-terminal end of the sRNA, while the percentage of the miRNAs had adenine (A), cytosine (C), or guanine (G) as the 5′ terminal nucleotide was less than 5% each. Sixty-eight to 73% of the miRNAs had C as 3′ terminal nucleotide, and 7–12% of the miRNAs had either A, G, or U as 3′ terminal nucleotide. This was similar for all treatments.

### Analysis of small RNAs homologous to the dsRNA insert

**Figure 4:**
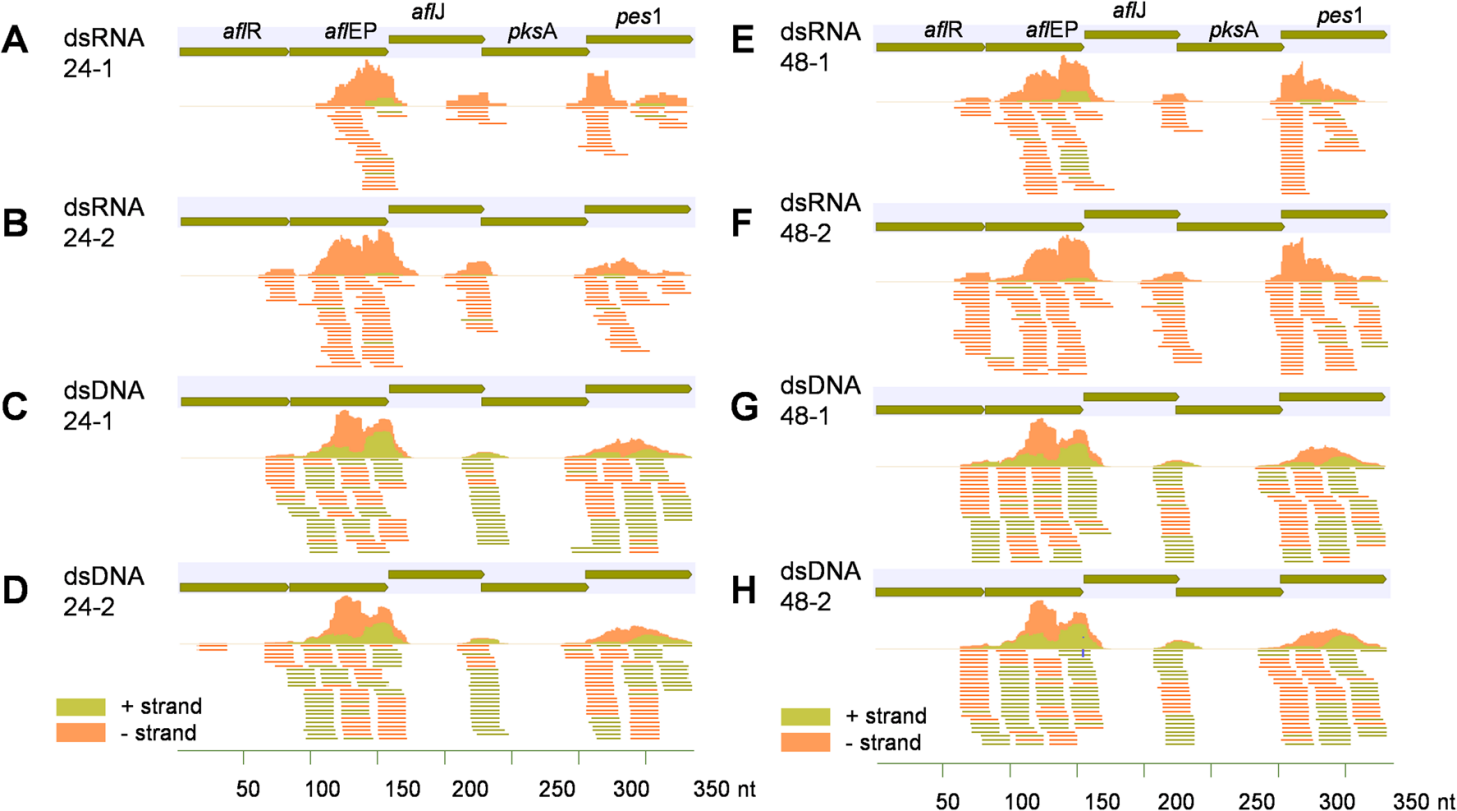
Small RNAs complementary to the RNAi-5x insert in libraries of dsRNA, and dsDNA treatments, after 24 and 48 h incubation post bombardment. (**A**) dsRNA24 h Rep 1. (**B**) dsRNA 24 h Rep 2. (**C**) dsDNA 24 h Rep 1. (**D**) dsDNA 24 h Rep 2. (**E**) dsRNA 48 h Rep 1. (F) dsRNA 48 h Rep 2. (**G**) dsDNA 48 h Rep 1. (**H**) dsDNA 48 h Rep 2.

The mappable reads that did not map to miRbase or the *A. duranensis* and *A. ipaënsis* genomes, were mapped to the dsRNA insert, to identify siRNAs possibly derived from the insert in the dsRNA or dsDNA treatments. There were 529 different siRNA types identified in the libraries of dsRNA and dsDNA treatments at both incubation periods (24 h and 48 h), that were not present in the control libraries. These 529 siRNAs mapped to the dsRNA insert and had a total abundance of 1619 reads, and all reads had perfect matching except three that showed a single mismatch in dsDNA at 48 h (Supplementary Table S3, Fig. 4A–H). In the libraries of dsRNA and dsDNA treated samples, more siRNA types were observed, and in higher abundance after 48 h incubation than after 24 h incubation (p = 4.18E^-05^) (Fig. 4A–H). The majority of the siRNAs types were present in the dsDNA treatments, regardless of incubation period (Supplementary Table S3, Table 3, Fig. 4A–H). The RNAi-5x insert as dsRNA generated mostly negative strand siRNAs (9:1 ratio), whereas dsDNA generated positive and negative strand sRNAs in similar proportion (5:5 ratio) (Fig. 4A–H). However, application of dsDNA resulted in a significantly higher (p ≤ 0.036) total amount of negative strand generated siRNAs than application of dsRNA, because more siRNAs were generated from the dsDNA (Fig. 4).

**Figure 5:**
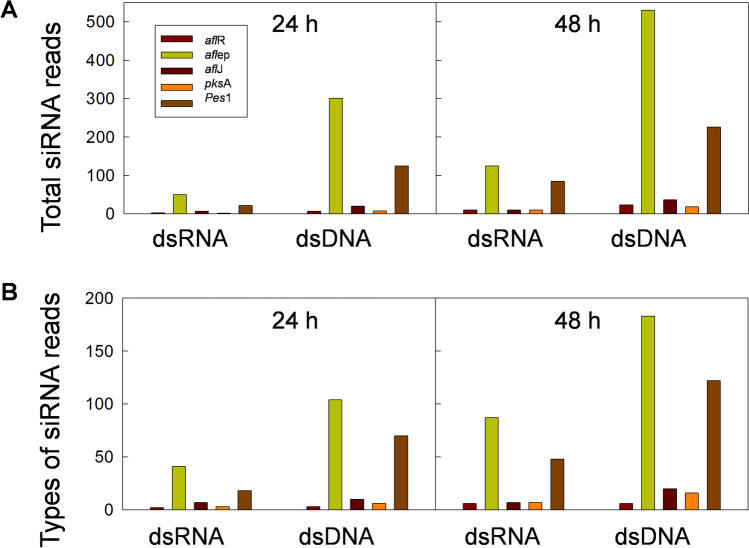
Small RNAs complementary to the RNAi-5x insert in libraries of dsRNA, and dsDNA treatments, after 24 and 48 h incubation post bombardment. (**A**) siRNA abundance per treatment. (**B**) siRNA types generated from the RNAi-5x insert per library.

We also identified siRNAs that were exclusively present in the libraries from the dsRNA treatment or the dsDNA treatment, as well as those that were present in both (Supplementary Table S3, Table 4). There were 132 siRNAs only in the dsRNA treatment that mapped without mismatches to the dsRNA insert, at either or both incubation periods. Of these, 25 were present only at 24 h, 86 only at 48 h, and 21 siRNAs at both incubation periods (Fig. 5A). There were 335 siRNAs exclusively present in the libraries of the dsDNA at either or both inoculation periods, of which 45 only at 24 h, 176 only at 48 h, and 114 at both 24 and 48 h (Fig. 5A). When considering insert- or plasmid-derived siRNAs with a minimum read count of 10 reads in at least one library, there were 14 siRNAs identified, one exclusively in the dsRNA treatment, seven only in the dsDNA treatment, and six siRNAs in both the dsRNA and the dsDNA treatment (Table 4). Most of the siRNA types found mapped to *afl*ep (271 siRNA types), followed by *pes1* (1,185 siRNA types); the least mapped to *afl*R (13 siRNA types) (Figs. 4, 5B). The majority of the siRNAs mapping without mismatch to the dsRNA insert had guanine as the 5′-terminal end, the percentages ranging from 28 to 41%. At the 3′-terminal end, cytosine was the most predominant. This was similar for the dsRNA and the dsDNA treatments at both incubation periods.

**Table 4:** Small RNAs complementary to the RNAi-5x insert in libraries of control, dsRNA, and dsDNA treatments, after 24 and 48 h incubation, with a minimum read count of 10 in at least one library.

sRNA	Gene	sRNA sequence	Length	Control 24 h	Control 48 h	dsRNA 24 h	dsRNA 48 h	dsDNA 24 h	dsDNA 48 h
151,424	*afl*ep	UGUUUCUGGUGGCAUUGGACC	21	0	0	0	0	4	10
112,565	*afl*ep	UCACGGACGAAUCCCCUGCAUC	22	0	0	0	0	6	11
98,716	*afl*ep	UCUUGUCAUCUCUACAGCCAUUC	23	0	0	0	0	8	12
101,788	*afl*ep	GCAUUGGACCGUCUUGUCAUC	21	0	0	0	0	6	13
65,440	*afl*ep	UCUUGUCAUCUCUACAGCCAUU	22	0	0	0	0	15	15
38,032	*afl*ep	GUCUUGUCAUCUCUACAGCCA	21	0	0	0	0	18	38
38,321	*afl*ep	ACCGUCUUGUCAUCUCUACAGCCA	24	0	0	0	2	17	37
106,715	*afl*ep	UCCCCAGAUCACGGACGAAUCCCC	24	0	0	4	12	1	1
113,005	*afl*ep	CCCCAGAUCACGGACGAAUCCC	22	0	0	0	1	6	11
128,820	*afl*ep	CCCAGAUCACGGACGAAUCCCC	22	0	0	1	1	4	11
61,031	*afl*ep	CCCAGAUCACGGACGAAUCCC	21	0	0	1	2	14	18
61,878	*afl*ep	GGACGAAUCCCCUGCAUCUACG	22	0	0	1	0	10	23
182,477	*pks*A	AGGCCACGGUAGGGAGG	17	0	0	1	11	0	0
143,562	*pes*1	ACUCUGGAAUUGACAUUUCGCGUA	24	0	0	0	0	4	10

### Validation of sRNAs by stemloop qRT-PCR

QuantiTect One-step real time PCR approximated a negative linear correlation with the Log_10_ of the template concentration within 66.667 ng and 0.667 pg, the latter corresponding to approximately 62 million molecules of template. In reactions without template, the derivatives of the melting curves had a maximum at 79 °C, but this was distinguishable from samples with templates that had maxima at 81–83 °C.

Small RNAs corresponding to *afl*R and *afl*J were not detected in any of the samples. Small RNAs of *pks*A were detected both at 24 h and 48 h incubation samples of dsDNA treatment; the estimated amount of *pks*A template in these samples was 0.06 pg. No statistically significant differences were observed between the 24 h and 48 h samples. The miR166a, which was present in high abundance (> 100.000 reads) in all treatments, was detected using qRT-PCR in all the treatments and all the biological samples. For this miRNA the estimated detection was also 0.06 pg of template. Melting curves for miR166 had different shape for positive and negative controls. The T-test comparison between samples showed significant differences between miR166a expression in dsDNA and dsRNA at 48 h, p ≤ 0.057; and between the control and dsRNA treatments at 48 h, p ≤ 0.019 (Table 5).

**Table 5:** Validation of sRNAs by stemloop qRT-PCR.

siRNA/reference miRNA	sRNA sequencing					
	Abundance control 24 hpb	Abundance control 48 hpb	Abundance dsRNA 24 h	Abundance dsRNA 48 h	Abundance dsDNA 24 h	Abundance dsDNA 48 h
siRNA_186906	0	0	1	0	4	6
siRNA_141931	0	0	0	0	4	9
siRNA_568285	0	0	0	0	1	3
gma-miR166a-3p	432,414	421,684	387,193	363,170	466,733	393,277

siRNA/reference miRNA	Stemloop qRT-PCR					
	Ct Control 24 hpb	Ct Control 48 hpb	Ct dsRNA 24 h	Ct dsRNA 48 h	Ct dsDNA 24 h	Ct dsDNA 48 h
**siRNA_186906**						
Rep 1*	33.30	33.18	33.75	33.08	33.17	33.62
**siRNA_186906**						
Rep 1*	33.30	33.18	33.75	33.08	33.17	33.62
Rep 2*	33.55	32.95	33.30	33.04	33.69	33.50
**siRNA_141931**						
Rep 1*	21.48	21.48	22.30	21.37	21.65	22.49
Rep 2*	22.35	21.12	21.81	21.62	22.19	22.35
**siRNA_568285**						
Rep 1*	38.65	38.35	38.08	37.26	39.27	38.68
Rep 2*	39.29	39.14	38.26	37.88	38.67	37.95
**gma-miR166a-3p**						
Rep 1*	26.93	26.83	27.36	27.12	26.99	29.71
Rep 2*	27.07	27.24	27.10	27.92	28.78	29.17

^*^Rep: biological replicate.

## Discussion

The results of this study show the biogenesis of siRNAs derived from all five gene fragments in the RNAi-5x insert that targets for silencing aflatoxin-synthesis genes. The analyses were performed after 24 and 48 h of applying RNAi-trigger signals (dsRNA and dsDNA) in peanut leaves. The effect of the treatments on plant miRNAs related to disease resistance, AGO1 and TAS3 are also analyzed. Our research is the first study that compares siRNAs generated from a plasmid (dsDNA) and dsRNA construct in *in-vitro* peanut plants. Some studies on sRNA in peanut plants described natural miRNA populations and their functions^26,27,30^, or were focused on miRNA populations during embryogenesis and early pod development^28,29^. Other studies successfully targeted the control of plant disease by using multiple RNAi fragments, yet these fragments had single gene targets for silencing^8–12^. None of those studies targeted multiple genes in the metabolic pathway of a pathogen, as in this case the RNAi-5x insert that consists of fragments of five genes involved in aflatoxin biosynthesis of *Aspergillus flavus*.

The aim of this study was to obtain a better understanding of the early steps of the RNAi mechanism in peanut plants when targeting aflatoxin synthesis genes of the pathogen, and the potential differences in efficacy of generating siRNAs whether an RNAi-5x insert is introduced in the plant as dsRNA or dsDNA. In previous studies^13,15^, we obtained transformed peanut plants expressing RNAi-5x that prevented aflatoxin accumulation in seeds. Information on the types of siRNAs generated from the RNAi-5x insert, their abundance, and the timeframe in which the RNAi process is initiated, was required to provide insight on the involvement of the RNAi-5x construct.

There are several requirements for RNAi to be an effective method to silence target mRNA, i.e*.*: DICER enzymes and AGO proteins involved in RNAi need to be active to generate siRNAs from the dsRNA and to load siRNAs onto AGO to form the RISC complex^17,19^. With this study, we were able to show that many siRNAs were generated from the RNAi-5x insert within 24 h after being introduced in the peanut leaves. The majority of the sRNAs were 20–24 nt in length, which is typical for DICER-processed sRNAs^18–20^, and has been previously reported in peanut^15,26–30^. However, we observed significantly higher abundance of 24-nt sRNAs when dsDNA was used, indicating that dsDNA favored activity of DCL3^31^. Furthermore, 21 and 22-nt sRNAs are in general associated with AGO1, AGO2, AGO7, and AGO10, while 24-nt sRNAs are generally associated with AGO4, AGO6, and AGO9^32,33^. The 5′-terminal end of the sRNAs was mostly uridine, indicating preferential loading onto AGO1^34^. Our results also show that siRNAs were generated from the introduced dsRNA insert, and that the majority of the siRNAs mapped to *afl*ep, followed by *pes*1, while fewer mapped to *afl*R. Since the siRNAs that mapped the RNAi-5x insert showed high abundance on specific areas of the insert, both for dsRNA and for dsDNA treatments, the sRNAs were interpreted as being generated de novo; a uniform coverage would be expected if they had originated from dsRNA degradation. In our previous study, we had observed a quantitative increase in the amount of RNA duplexes over time^25^.

Another requirement for RNAi to be successful in silencing is that siRNAs be generated in a relatively short timeframe, and that the generated siRNAs be active for an extended period of time^34–37^. The results of this study showed that longer incubation led to the generation of more siRNAs. In both treatments, dsRNA and dsDNA, more and higher abundance of siRNA types were generated from the dsRNA insert at 48 h than at 24 h; in most cases twice as many siRNAs at 48 h, and up to twice as many siRNA types at 48 h. In this study, a Gene Gun was used to ensure delivery of the RNAi signal to the peanut plant, and thus avoiding uncertainties of loss or degradation of sRNAs when using different application methods such as soil drench or spraying the foliage. In a different experiment of our RNAi research^25^, Dicer-substrate siRNAs (dsiRNAs) were applied to the top of the peanut plant, after wounding. We were able to demonstrate that the dsiRNA moved systemically from the application point through the vascular system to new auxiliary shoots, flowers, and newly formed pegs, within 15 days after treatment. The dsiRNA was detected up to 60 days after introduction to *in-vitro* peanut plants. Further research is needed to determine efficient application methods and the length of time siRNAs generated from the introduced dsRNA insert will remain and multiply in peanut plants.

RNAi-mediated host-induced gene silencing involves the genetic transformation with a construct consisting of a promoter and the DNA sequence of a target gene as an inverted repeat, separated by an intron^17,19^. In contrast, transient gene silencing, involves the introduction of double-stranded RNA sequence of a target gene, usually without promoter and this process does not involve transformation. In our study, we introduced the 393-bp dsRNA insert as dsRNA and as plasmid dsDNA and compared the siRNAs generated. More siRNA types were observed and in higher abundance after dsDNA treatments at both incubation periods, 24 and 48 h. Since dsRNA insert is driven by the 35S promoter in dsDNA^13,15^, dsRNA could have been generated not only from transient expression, but also from few transformed cells after the particle bombardment; this could have resulted in significantly higher type and abundance of siRNAs observed in this treatment. Another factor that could have contributed to higher abundance is the possible generation of mRNA and its degradation or sRNA turnover^35–37^.

There is limited small RNA sequence information on peanut available in databases, which is a limiting factor in annotating sRNAs. Consistent with previous reports^15,26,27^ a large percentage of the small RNAs found in the different libraries did not map to the peanut genome, miRbase, or the dsRNA insert, which could be due to unavailable sequence and annotation information of cultivated peanut. There is insufficient information available on the enzymes involved in RNAi in peanut, and more information is also needed on the targets and functions of miRNAs in this species. In this study, we observed a significant (p ≤ 0.05) down-regulation of miR399 and up-regulation of miR482 in leaves treated with dsRNA insert as dsDNA compared to the control. The micro RNA, miR399, is related to phosphorous nutrition and interaction of plants with fungi^38,39^ whereas miR482 belongs to the miR482/2,118 family that targets resistance genes in plants^40^. Whether the expression of miR399 and miR482 can provide an additional layer of protection against aflatoxigenic *Aspergillus* invading peanuts is a subject for further research. Knowledge on the functions and targets of the miRNAs would provide important information on possible secondary effects the construct can have that may be useful or detrimental to normal essential functions in the plant.

High consistency was observed between biological samples in the one-step stemloop qRT-PCR. The low temperature maximum observed in the derivative of the melting curves was probably generated by the stem-loop structure of the primers used for reverse transcription. In the one-step stemloop qRT-PCR, *pks*A siRNAs were detected only in the dsDNA treatment and was not detected in the control or in dsRNA treatment samples. Other RNAi-insert specific siRNAs were not detected, from the small RNA sequencing results we can infer that their abundance was below the threshold of detection in the qRT-PCR reactions. It is interesting to notice that the level of expression of miR166a at 48 h was significantly higher for the dsDNA treatment than the dsRNA (p ≤ 0.057) and also higher in the control treatment, than dsRNA (p ≤ 0.019). A similar trend was observed with the high throughput sRNA sequencing, though in that case the differences were not statistically significant. In these experiments, the one-step stemloop qRT-PCR detected as low as 25,000 molecules per microliter (6 million per reaction), thus, it is not surprising that no amplification was detected for some dsDNA and dsRNA-specific siRNAs tested. However, in the case of miR166a, sequencing results showed between 445,000 and 528,000 reads for miR166a in all the treatments, and the one-step stemloop qRT-PCR consistently detected the template in all samples.

Altogether, we identified siRNAs that were generated from all five gene fragments, with the majority of siRNAs mapping to *afl*ep and siRNAs expressed as early as 24 h after treatment, and increasing even more after 48 h. These results bring us closer to the understanding of RNAi in peanut in general, and specifically, on how to utilize the RNAi technology to reduce aflatoxin accumulation in peanut.

## Supplementary information

Supplementary information.
