# Supplementary material for: Analysis of small RNA populations generated in peanut leaves after exogenous application of dsRNA and dsDNA targeting aflatoxin synthesis genes
Source: Sci Rep. 2020 Aug 14;10:13820. doi: 10.1038/s41598-020-70618-6 (PMC7427784; doi:10.1038/s41598-020-70618-6)
Supplement: Supplementary file 1 — Supplementary information. [file 41598_2020_70618_MOESM1_ESM.pdf]

Analysis of small RNA populations generated in peanut leaves after exogenous application of dsRNA and dsDNA targeting aflatoxin synthesis genes

Imana L. Power[1,2*], Paola C. Faustinelli[1], Valerie A. Orner[1], Victor S. Sobolev[1], and Renee S. Arias[1]

[1] United States Department of Agriculture, Agricultural Research Service, National Peanut Research Laboratory (NPRL), Dawson, Georgia, United States of America

[2]Current address: Center for Agricultural Research in Suriname (CELOS), Paramaribo, Suriname

* Corresponding author

Email: imanapower@yahoo.com (IP)

Supplementary Table S1

miRNA Family	Control 24 h	dsRNA 24 h	dsDNA 24 h	Total members 24 h	Control 48 h	dsRNA 48 h	dsDNA 48 h	Total members 48 h
miR156	17,265	12,852	9,723	21	12,556	13,124	9,619	22
miR159	28,058	18,256	14,092	4	20,692	21,734	14,582	4
miR160	148	185	147	3	128	158	131	1
miR162	484	693	633	2	513	480	488	3
miR164	38	27	21	3	38	58	22	2
miR166	491,637	445,469	528,400	15	484,086	425,470	460,811	15
miR167	10,916	7,316	7,462	10	8,179	6,947	6,736	10
miR168	3,417	4,982	3,316	3	3,074	3,752	2,498	3
miR169	65	115	129	5	65	100	131	4
miR171	192	184	183	6	168	203	202	7
miR172	389	491	382	4	418	465	434	4
miR319	732	507	446	4	651	634	465	5
miR390	77	130	112	2	83	106	91	2
miR393	456	250	275	4	332	240	265	5
miR394	858	1,070	727	3	689	1,048	774	4
miR395	217	528	228	10	302	513	269	10
miR396	64,204	97,325	88,918	8	80,836	92,803	86,070	8
miR397	79	29	30	1	62	29	39	1
miR398	50	91	116	2	326	96	265	2
miR399	80	47	29	2	66	69	30	3
miR401	0	0	0	0	3	13	2	1
miR408	4,266	2,233	2,247	3	2,447	2,176	1,693	3
miR472	22	12	6	1	20	18	10	1
miR477	30	65	49	2	41	56	48	3
miR479	4	18	9	1	6	25	18	1
miR482	19,835	29,155	31,680	3	22,127	23,573	31,828	4
miR821	15	10	5	1	19	10	10	1
miR862	232	337	236	1	240	283	293	2
miR894	91	83	68	1	136	94	74	1
miR952	0	0	0	0	5	14	3	1
miR1065	0	0	0	0	9	6	3	1
miR1103	32	15	4	2	30	54	12	1
miR1122	0	0	0	0	3	13	3	1
miR1432	10	2	1	1	8	18	4	1
miR1507	16,493	18,352	20,431	2	16,032	16,115	17,303	2
miR1511	3,027	3,447	2,463	3	2,947	3,687	2,521	3
miR1851	21	15	17	1	4	11	2	1
miR2111	8,004	6,942	8,595	4	15	10	16	1
miR2118	0	0	0	0	8,869	6,088	8,379	3
miR2275	0	0	0	0	3	8	2	1
miR2592	47	15	6	1	55	127	19	1
miR2603	0	0	0	0	10	19	2	1
miR2673	43	44	33	1	31	64	33	1
miR2916	2,957	1,829	1,892	2	6,436	6,967	6,450	2
miR2931	0	0	0	0	1	6	1	1
miR3440	0	0	0	0	1	8	1	1
miR3508	2,494	1,448	1,356	1	1,623	1,241	1,100	1
miR3509	849	1,003	1,149	4	866	803	1,042	4
miR3511	9	1	3	1	9	3	3	1
miR3512	9	4	6	1	23	23	15	1
miR3513	461	483	464	2	450	455	425	2

All data have been deposited in FigShare: doi: **10.6084/m9.figshare.11771628**. The mapping coordinates are available in the different “**comp_6492_**” files.

miR3514	3,303	4,196	4,214	1	3,273	2,881	3,374	1
miR3515	25	26	47	2	60	24	74	2
miR3516	27	13	15	1	25	10	11	1
miR3518	181	224	174	2	166	293	175	2
miR3520	31	24	29	3	21	14	23	2
miR3627	16	20	11	1	35	37	13	1
miR3701	12	5	3	1	6	14	5	1
miR4236	27	13	4	1	28	73	9	1
miR4243	7	4	4	1	11	22	2	1
miR4382	8	2	0	1	1	10	3	1
miR4995	0	0	0	0	4	17	4	1
miR5037	9	13	5	1	8	19	6	1
miR5054	22	6	3	1	21	21	6	1
miR5059	0	0	0	0	11	21	7	1
miR5083	19	3	2	1	12	9	3	1
miR5141	157	106	85	2	166	108	101	2
miR5167	19	11	2	1	16	21	5	1
miR5211	8	10	15	1	44	35	46	1
miR5223	8	5	3	1	9	10	3	1
miR5266	0	0	0	0	2	19	2	1
miR5291	13	1	2	1	16	17	7	1
miR5294	11	3	4	1	10	12	3	1
miR5298	0	0	0	0	4	11	1	1
miR5303	0	0	0	0	4	16	4	1
miR5368	7,742	7,740	6,830	2	8,902	13,475	9,403	2
miR5381	399	270	74	1	347	921	121	1
miR5493	8	3	0	1	32	21	8	1
miR5652	200	47	22	1	199	338	82	1
miR5724	218	46	24	1	136	284	49	1
miR6025	88	41	48	1	130	26	39	1
miR6300	15	6	2	1	22	35	7	2
miR6475	0	0	0	0	6	8	2	1
miR6478	17	7	2	1	26	36	6	1
miR7485	0	0	0	0	2	19	3	1
miR7504	0	0	0	0	4	7	0	1
miR7528	0	0	0	0	11	7	1	1
miR7530	0	0	0	0	3	12	1	1
miR7693	0	0	0	0	2	7	0	1
miR8154	21	10	4	1	16	116	6	1
miR8155	224	55	32	1	8	10	5	1
miR8175	0	0	0	0	171	167	42	1
miR8572	0	0	0	0	5	12	1	1
miR8681	0	0	0	0	2	9	2	1
miR9410	10	4	2	1	3	17	3	1
miR9475	0	0	0	0	7	15	2	1
miR9674	0	0	0	0	4	20	3	1

Supplementary Table S2

**Reference miRNA**	**miRNA Sequence**	**Length**	**Abundance Control 24 h**	**Abundance dsRNA 24 h**	**Abundance dsDNA 24 h**	**Log2 (dsRNA/ Control)**	**Log2 (dsDNA/ Control)**	***P***
smo-MIR1103-p3_1ss6CT	CCUCCUGGGAAGUCC	15	25	11	4	-1.16	**-2.80**	8.19E-02
sbi-MIR5381-p5_1ss11AG	CUCGGCGCCAGGAUC	15	399	270	74	-0.56	**-2.43**	6.38E-02
sbi-MIR171b-p5_1ss16GT	CGCGACGGGGUAUUGU	16	10	2	2	**-2.11**	**-2.22**	3.11E-02
ptc-miR166p_L+2R-2	UCUCGGACCAGGCUCCAUUCC	21	10	4	2	-1.31	**-2.24**	3.48E-02
osa-MIR5083-p3_1ss15GT	AUCCUUCUGAGCCUU	15	19	3	2	**-2.54**	**-3.47**	4.58E-02
mtr-MIR2592bi-p5_1ss8TC	AUUCCCACUGUCCCUGU	17	47	15	6	-1.61	**-2.92**	4.60E-02
gma-miR6300_L-3	GUUGUAGUAUAGUGG	15	15	6	2	-1.20	**-3.07**	7.66E-02
gma-MIR4382-p3_1ss9AC	GGGGAAGUCGGAAUA	15	8	2	0	**-2.10**	**-12.90**	1.34E-03
bra-MIR5724-p3_1ss1TA	AAUCAUGUGGGCUCU	15	218	46	24	**-2.23**	**-3.19**	9.35E-03
ath-miR8175_L-2	UCCCCGGCAACGGCGCCA	18	224	55	32	**-2.03**	**-2.80**	5.17E-02
ath-MIR5652-p5_1ss7TC	ACAAUACCGGGCUCA	15	200	47	22	**-2.10**	**-3.15**	1.35E-02
aly-MIR4236-p5_1ss5CG	GACGGAAGGGCACCA	15	27	13	4	-1.04	**-2.59**	9.42E-02
ahy-miR160-5p	UGCCUGGCUCCCUGAAUGCCA	21	1	10	5	**3.15**	**2.15**	2.81E-02
ahy-miR156b-3p	GCUCUCUAAGCUUCUGUCAUC	21	5	17	19	1.91	**2.05**	4.32E-02
ahy-MIR398-p5_1ss9CA	GGAGUGAAACUGAGAACACAAAG	23	12	44	50	1.92	**2.11**	4.03E-02

**Reference miRNA**	**miRNA Sequence**	**Length**	**Abundance Control 48 h**	**Abundance dsRNA 48 h**	**Abundance dsDNA 48 h**	**Log2 (dsRNA/ Control)**	**Log2 (dsDNA/ Control)**	***P***
zma-miR395a-3p_L-5R-1	GUGUUUGGGGGAACU	15	3	10	0	1.77	**-11.50**	9.53E-05
ptc-miR6478_R-5_1ss10GA	CCGACCUUAACUCAGU	16	26	36	6	0.50	**-2.12**	5.54E-02
osa-miR7693-3p_L-6R-1_1ss19GA	CAUCGAUGAAGAACG	15	2	7	0	**2.05**	**-10.67**	1.92E-03
mtr-miR5266_L-4R-1_1ss10TA	GGGACAGUCUGGGGC	15	2	19	2	**3.57**	**0.61**	8.39E-03
mtr-MIR5298b-p3_1ss15GT	AAGAUGAAAAGGACU	15	4	11	1	1.47	**-2.00**	1.17E-02
lja-MIR7530-p5_1ss9TC	CCCUCUCCCCCUUCCU	16	3	12	1	**2.10**	-1.57	7.91E-03
gra-MIR7504k-p3_1ss9AC	UGAAAAUCCGGAGGA	15	4	7	0	0.80	**-12.03**	6.15E-03
gma-miR399a_L-1R-2	GCCAAAGGAGAGUUGCCC	18	6	8	1	0.32	**-3.11**	2.24E-02
ghr-MIR7485-p5_1ss14TC	CCUCUUUCCCUUCCU	15	2	19	3	**3.41**	0.97	2.49E-02
cpa-MIR8154-p3_1ss8CT	CCCUCUCUCUCCUCC	15	16	116	6	**2.84**	-1.45	9.27E-02
bol-MIR9410-p3_1ss5TG	CUUUGCAGACGACUU	15	3	17	3	**2.66**	0.08	4.90E-03
atr-MIR8572-p5_1ss13TC	CCCCAGUGGAGUCGC	15	5	12	1	1.35	**-2.31**	3.23E-02
aly-MIR4243-p3_1ss12AC	UGAGAGCAUGCCUGUC	16	11	22	2	1.03	**-2.59**	5.09E-02
ahy-miR156b-3p_R+1_2ss9AT10GT	GCUCUCUAUUCUUCUGUCAUCU	22	11	3	3	-1.75	**-2.04**	4.65E-02

All data have been deposited in FigShare: doi: **10.6084/m9.figshare.11771628**. The mapping coordinates are available in the different "**comp_6492_**" files.

Supplementary Table S3

siRNA	Strand	Gene	Start	Sequence	Length	Control 24 h	Control 48 h	dsRNA 24 h	dsRNA 48 h	dsDNA 24 h	dsDNA 48 h
siRNA_1487746	-	*afl* R	60	GGUCUUGCCUGUCAAUACAUGGUCGUAU	28	0	0	0	1	0	0
siRNA_962224	-	*afl* R	60	GGUCUUGCCUGUCAAUACAUGGUCGUA	27	0	0	0	2	0	0
siRNA_1277017	-	*afl* R	65	UGCCUGUCAAUACAUGGUCGUAU	23	0	0	0	2	0	0
siRNA_265665	-	*afl* R	65	UGCCUGUCAAUACAUGGUCGU	21	0	0	0	0	1	6
siRNA_186906	-	*afl* R	65	UGCCUGUCAAUACAUGGUCGUA	22	0	0	1	0	4	6
siRNA_1549296	-	*afl* R	66	GCCUGUCAAUACAUGGUCGUA	21	0	0	0	0	0	1
siRNA_2913232	+	*afl* R	67	CCUGUCAAUACAUGGUCGUAU	21	0	0	0	0	0	1
siRNA_3355126	-	*afl* R	69	UGUCAAUACAUGGUCGU	17	0	0	0	1	0	0
siRNA_852019	-	*afl* R	69	UGUCAAUACAUGGUCGUAU	19	0	0	1	1	0	0
siRNA_543477	-	*afl* R	69	UGUCAAUACAUGGUCGUA	18	0	0	0	2	0	0
siRNA_2850551	-	*afl* R	73	AAUACAUGGUCGUAUUUGUGAC	22	0	0	0	0	1	0
siRNA_606850	+	*afl* R	73	AAUACAUGGUCGUAUUUGUGAC	22	0	0	0	0	0	3
siRNA_322442	+	*afl* R	74	AUACAUGGUCGUAUUUGUGAC	21	0	0	0	0	0	6
siRNA_4005125	+	*afl* ep	81	GUCGUAUUUGUGACCAUGUUUC	22	0	0	0	0	0	1
siRNA_2068616	+	*afl* ep	82	UCGUAUUUGUGACCAUGUUUCU	22	0	0	0	0	0	1
siRNA_797920	+	*afl* ep	82	UCGUAUUUGUGACCAUGUUUC	21	0	0	0	0	1	2
siRNA_1666418	+	*afl* ep	83	CGUAUUUGUGACCAUGUUUCUGG	23	0	0	0	0	0	1
siRNA_409172	+	*afl* ep	83	CGUAUUUGUGACCAUGUUUCU	21	0	0	0	0	1	4
siRNA_965707	+	*afl* ep	85	UAUUUGUGACCAUGUUUCUGG	21	0	0	0	0	0	2
siRNA_2776872	-	*afl* ep	86	AUUUGUGACCAUGUUUCUGG	20	0	0	0	1	0	0
siRNA_1466180	-	*afl* ep	91	UGACCAUGUUUCUGGUGGCAUUGGACCGUCUUG	33	0	0	0	1	0	0
siRNA_2919449	-	*afl* ep	92	GACCAUGUUUCUGGUGGCAUUGGACCGUC	29	0	0	0	1	0	0
siRNA_1345076	-	*afl* ep	93	ACCAUGUUUCUGGUGGCAUUGG	22	0	0	0	0	0	1
siRNA_1628558	-	*afl* ep	94	CCAUGUUUCUGGUGGCAUUGGA	22	0	0	0	0	0	1
siRNA_932133	-	*afl* ep	94	CCAUGUUUCUGGUGGCAUUGG	21	0	0	0	0	0	2
siRNA_1665611	-	*afl* ep	95	CAUGUUUCUGGUGGCAU	17	0	0	0	2	0	0
siRNA_2535926	+	*afl* ep	95	CAUGUUUCUGGUGGCAUUGGA	21	0	0	0	0	0	1
siRNA_1716938	+	*afl* ep	95	CAUGUUUCUGGUGGCAUUGGACCG	24	0	0	0	0	0	1
siRNA_1132164	+	*afl* ep	95	CAUGUUUCUGGUGGCAUUGGACC	23	0	0	0	0	1	1
siRNA_2647757	+	*afl* ep	96	AUGUUUCUGGUGGCAUUGGAC	21	0	0	0	0	0	1
siRNA_571833	-	*afl* ep	96	AUGUUUCUGGUGGCAUUGGAC	21	0	0	0	0	2	2
siRNA_333493	+	*afl* ep	96	AUGUUUCUGGUGGCAUUGGACC	22	0	0	0	0	3	3
siRNA_4122314	+	*afl* ep	97	UGUUUCUGGUGGCAUUGGACCGU	23	0	0	0	0	0	1
siRNA_953716	+	*afl* ep	97	UGUUUCUGGUGGCAUUGGACCGUC	24	0	0	0	0	1	1
siRNA_151424	+	*afl* ep	97	UGUUUCUGGUGGCAUUGGACC	21	0	0	0	0	4	10
siRNA_1069167	-	*afl* ep	98	GUUUCUGGUGGCAUUGGACCG	21	0	0	0	1	0	0
siRNA_4294981	+	*afl* ep	98	GUUUCUGGUGGCAUUGGACCG	21	0	0	0	0	0	1
siRNA_1166783	-	*afl* ep	98	GUUUCUGGUGGCAUUGGACCGUCU	24	0	0	0	1	0	1

siRNA_1044318	+	*afl* ep	99	UUUCUGGUGGCAUUGGACCGU	21	0	0	0	0	1	0
siRNA_2705417	+	*afl* ep	99	UUUCUGGUGGCAUUGGACCGUCUU	24	0	0	0	0	0	1
siRNA_1877729	+	*afl* ep	99	UUUCUGGUGGCAUUGGACCGUCU	23	0	0	0	0	0	1
siRNA_304219	+	*afl* ep	99	UUUCUGGUGGCAUUGGACCGUC	22	0	0	0	0	2	5
siRNA_3387393	+	*afl* ep	100	UUCUGGUGGCAUUGGACCGUCU	22	0	0	0	0	0	1
siRNA_2803742	+	*afl* ep	100	UUCUGGUGGCAUUGGACC	18	0	0	0	0	0	1
siRNA_3049623	-	*afl* ep	101	UCUGGUGGCAUUGGACCGUC	20	0	0	0	1	0	0
siRNA_2891403	-	*afl* ep	101	UCUGGUGGCAUUGGACCGUCUUG	23	0	0	0	1	0	0
siRNA_2273528	-	*afl* ep	101	UCUGGUGGCAUUGGACCGUCUU	22	0	0	0	0	0	1
siRNA_943160	+	*afl* ep	101	UCUGGUGGCAUUGGACCGUCU	21	0	0	0	0	0	2
siRNA_2822778	-	*afl* ep	103	UGGUGGCAUUGGACCGUCUUGU	22	0	0	0	1	0	1
siRNA_1010387	-	*afl* ep	103	UGGUGGCAUUGGACCGUCUUG	21	0	0	1	1	0	1
siRNA_3164902	-	*afl* ep	104	GGUGGCAUUGGACCGUCU	18	0	0	1	0	0	0
siRNA_1562453	-	*afl* ep	104	GGUGGCAUUGGACCGUCUUGUC	22	0	0	1	1	0	0
siRNA_1100669	-	*afl* ep	104	GGUGGCAUUGGACCGUCUUGUCA	23	0	0	0	0	0	1
siRNA_823228	-	*afl* ep	104	GGUGGCAUUGGACCGUCUUGU	21	0	0	0	0	1	1
siRNA_1668392	-	*afl* ep	104	GGUGGCAUUGGACCGUCUUGUCAU	24	0	0	0	0	0	2
siRNA_1983992	+	*afl* ep	105	GUGGCAUUGGACCGUCUUGUC	21	0	0	0	0	0	1
siRNA_1967267	-	*afl* ep	106	UGGCAUUGGACCGUCUUGUCA	21	0	0	0	0	1	0
siRNA_3716218	+	*afl* ep	106	UGGCAUUGGACCGUCUUGUCAUCU	24	0	0	0	0	0	1
siRNA_183999	+	*afl* ep	106	UGGCAUUGGACCGUCUUGUCAUC	23	0	0	0	0	4	7
siRNA_1645149	-	*afl* ep	107	GGCAUUGGACCGUCUUGUCAUCUCUACAGCCAU	33	0	0	1	0	0	0
siRNA_4238434	+	*afl* ep	107	GGCAUUGGACCGUCUUGUCAUCUCU	25	0	0	0	0	1	0
siRNA_1237865	-	*afl* ep	107	GGCAUUGGACCGUCUUGUCA	20	0	0	0	0	1	0
siRNA_2361749	+	*afl* ep	107	GGCAUUGGACCGUCUUGUCAUCUC	24	0	0	0	0	0	1
siRNA_922458	+	*afl* ep	107	GGCAUUGGACCGUCUUGUCAUC	22	0	0	0	0	0	2
siRNA_460499	-	*afl* ep	107	GGCAUUGGACCGUCUUGUCAUCUC	24	0	0	0	0	1	3
siRNA_2548946	+	*afl* ep	108	GCAUUGGACCGUCUUGUC	18	0	0	0	1	0	0
siRNA_3601008	-	*afl* ep	108	GCAUUGGACCGUCUUGUCAUC	21	0	0	0	0	0	1
siRNA_563205	-	*afl* ep	108	GCAUUGGACCGUCUUGUCAUCUCU	24	0	0	1	0	2	1
siRNA_1712847	+	*afl* ep	108	GCAUUGGACCGUCUUGUCAUCUCU	24	0	0	0	0	0	1
siRNA_101788	+	*afl* ep	108	GCAUUGGACCGUCUUGUCAUC	21	0	0	0	0	6	13
siRNA_1292307	+	*afl* ep	109	CAUUGGACCGUCUUGUCAUC	20	0	0	0	0	1	1
siRNA_1711633	+	*afl* ep	109	CAUUGGACCGUCUUGUCAUCU	21	0	0	0	0	0	2
siRNA_681181	+	*afl* ep	109	CAUUGGACCGUCUUGUCAUCUCU	23	0	0	0	0	0	3
siRNA_472111	+	*afl* ep	109	CAUUGGACCGUCUUGUCAUCUC	22	0	0	0	0	1	3
siRNA_1539866	-	*afl* ep	110	AUUGGACCGUCUUGUCAUCUCUA	23	0	0	0	1	0	0
siRNA_1620357	-	*afl* ep	110	AUUGGACCGUCUUGUCAUCUCUAC	24	0	0	0	0	0	1
siRNA_1708667	+	*afl* ep	110	AUUGGACCGUCUUGUCAUCUCU	22	0	0	0	0	0	1
siRNA_1407409	+	*afl* ep	110	AUUGGACCGUCUUGUCAUCUCUAC	24	0	0	0	0	0	1
siRNA_695892	-	*afl* ep	110	AUUGGACCGUCUUGUCAUCUC	21	0	0	0	1	0	1

siRNA_1131100	+	*afl* ep	110	AUUGGACCGUCUUGUCAUCUC	21	0	0	0	0	0	2
siRNA_378644	-	*afl* ep	110	AUUGGACCGUCUUGUCAUCUCU	22	0	0	0	0	2	3
siRNA_364675	+	*afl* ep	111	UUGGACCGUCUUGUCAUCUCU	21	0	0	0	0	1	4
siRNA_2076582	+	*afl* ep	112	UGGACCGUCUUGUCAUCUCUAC	22	0	0	0	0	0	1
siRNA_2925457	-	*afl* ep	113	GGACCGUCUUGUCAUCUCU	19	0	0	0	1	0	0
siRNA_3966005	-	*afl* ep	113	GGACCGUCUUGUCAUCUCUACAGCC	25	0	0	0	1	0	0
siRNA_1143158	-	*afl* ep	113	GGACCGUCUUGUCAUCUCUACAGCCA	26	0	0	1	1	0	0
siRNA_259636	-	*afl* ep	113	GGACCGUCUUGUCAUCUC	18	0	0	2	5	0	0
siRNA_1775651	-	*afl* ep	113	GGACCGUCUUGUCAUCUCUA	20	0	0	0	0	1	0
siRNA_1086357	-	*afl* ep	113	GGACCGUCUUGUCAUCUCUAC	21	0	0	0	1	0	1
siRNA_3078789	+	*afl* ep	113	GGACCGUCUUGUCAUCUCUACAGC	24	0	0	0	0	0	1
siRNA_503887	-	*afl* ep	113	GGACCGUCUUGUCAUCUCUACA	22	0	0	0	1	0	3
siRNA_4291716	-	*afl* ep	114	GACCGUCUUGUCAUCUCUAC	20	0	0	0	1	0	0
siRNA_2966606	-	*afl* ep	114	GACCGUCUUGUCAUCU	16	0	0	0	1	0	0
siRNA_2725207	-	*afl* ep	114	GACCGUCUUGUCAUCUCUACAGCCAUUC	28	0	0	0	0	1	0
siRNA_2112080	-	*afl* ep	114	GACCGUCUUGUCAUCUCUACAGCCA	25	0	0	0	0	0	1
siRNA_468696	-	*afl* ep	114	GACCGUCUUGUCAUCUCUACA	21	0	0	0	0	2	2
siRNA_1497235	-	*afl* ep	115	ACCGUCUUGUCAUCUCUACAGCCAU	25	0	0	0	1	0	0
siRNA_980533	-	*afl* ep	115	ACCGUCUUGUCAUCUCUAC	19	0	0	0	2	0	0
siRNA_2031198	-	*afl* ep	115	ACCGUCUUGUCAUCUCUACAGC	22	0	0	0	0	0	1
siRNA_729118	-	*afl* ep	115	ACCGUCUUGUCAUCUCUACAGCC	23	0	0	0	1	1	1
siRNA_1313024	-	*afl* ep	115	ACCGUCUUGUCAUCUCUACAG	21	0	0	0	0	0	1
siRNA_372012	+	*afl* ep	115	ACCGUCUUGUCAUCUCUACAGC	22	0	0	0	0	1	4
siRNA_38321	-	*afl* ep	115	ACCGUCUUGUCAUCUCUACAGCCA	24	0	0	0	2	17	37
siRNA_3161304	-	*afl* ep	116	CCGUCUUGUCAUCUCUACAGCC	22	0	0	0	0	0	1
siRNA_2682933	-	*afl* ep	116	CCGUCUUGUCAUCUCUACAGCCA	23	0	0	0	1	0	1
siRNA_986414	-	*afl* ep	116	CCGUCUUGUCAUCUCUACAGCCAU	24	0	0	1	1	0	1
siRNA_1529241	+	*afl* ep	116	CCGUCUUGUCAUCUCUACAGCC	22	0	0	0	0	0	1
siRNA_649423	+	*afl* ep	116	CCGUCUUGUCAUCUCUACAGC	21	0	0	0	0	1	1
siRNA_1112453	+	*afl* ep	116	CCGUCUUGUCAUCUCUACAGCCAUUCCC	28	0	0	0	0	0	1
siRNA_3717693	-	*afl* ep	117	CGUCUUGUCAUCUCUACAGCC	21	0	0	0	0	1	0
siRNA_2199939	+	*afl* ep	117	CGUCUUGUCAUCUCUACAGCC	21	0	0	0	0	0	1
siRNA_547008	+	*afl* ep	117	CGUCUUGUCAUCUCUACAGCCAUU	24	0	0	0	0	2	1
siRNA_546816	-	*afl* ep	117	CGUCUUGUCAUCUCUACAGCCAUU	24	0	0	0	0	1	2
siRNA_271224	-	*afl* ep	117	CGUCUUGUCAUCUCUACAGCCA	22	0	0	1	0	3	4
siRNA_4178587	-	*afl* ep	118	GUCUUGUCAUCUCUACAGCCAUUCCC	26	0	0	1	0	0	0
siRNA_2690639	-	*afl* ep	118	GUCUUGUCAUCUCUACA	17	0	0	0	1	0	0
siRNA_1796030	-	*afl* ep	118	GUCUUGUCAUCUCUACAGCCAUUCC	25	0	0	0	0	1	0
siRNA_2999896	+	*afl* ep	118	GUCUUGUCAUCUCUACAGCCAUU	23	0	0	0	0	0	1
siRNA_4217657	+	*afl* ep	118	GUCUUGUCAUCUCUACAGCCAU	22	0	0	0	0	0	1
siRNA_184654	-	*afl* ep	118	GUCUUGUCAUCUCUACAGCCAU	22	0	0	1	0	4	6

siRNA_129536	-	*afl* ep	118	GUCUUGUCAUCUCUACAGCCAUUC	24	0	0	0	0	6	9
siRNA_38032	-	*afl* ep	118	GUCUUGUCAUCUCUACAGCCA	21	0	0	0	0	18	38
siRNA_3451361	-	*afl* ep	119	UCUUGUCAUCUCUACAGCCAUUCCCCAGA	29	0	0	1	0	0	0
siRNA_1740538	-	*afl* ep	119	UCUUGUCAUCUCUACAGCC	19	0	0	1	0	0	1
siRNA_563026	-	*afl* ep	119	UCUUGUCAUCUCUACAGCCA	20	0	0	0	1	1	2
siRNA_646336	+	*afl* ep	119	UCUUGUCAUCUCUACAGCCAUU	22	0	0	0	0	1	2
siRNA_133405	-	*afl* ep	119	UCUUGUCAUCUCUACAGCCAUUCC	24	0	0	0	0	6	8
siRNA_96243	-	*afl* ep	119	UCUUGUCAUCUCUACAGCCAU	21	0	0	1	2	9	9
siRNA_98716	-	*afl* ep	119	UCUUGUCAUCUCUACAGCCAUUC	23	0	0	0	0	8	12
siRNA_65440	-	*afl* ep	119	UCUUGUCAUCUCUACAGCCAUU	22	0	0	0	0	15	15
siRNA_2625001	-	*afl* ep	120	CUUGUCAUCUCUACAGCCAUU	21	0	0	0	0	1	0
siRNA_3693027	+	*afl* ep	120	CUUGUCAUCUCUACAGCCAUU	21	0	0	0	0	1	0
siRNA_1195425	+	*afl* ep	120	CUUGUCAUCUCUACAGCCAUUCCC	24	0	0	0	0	1	1
siRNA_1374998	-	*afl* ep	120	CUUGUCAUCUCUACAGCCAUUC	22	0	0	0	0	0	2
siRNA_972139	-	*afl* ep	121	UUGUCAUCUCUACAGCCA	18	0	0	0	2	0	0
siRNA_2794754	+	*afl* ep	121	UUGUCAUCUCUACAGCCAUUCCCC	24	0	0	0	0	0	1
siRNA_1228165	+	*afl* ep	121	UUGUCAUCUCUACAGCCAUUC	21	0	0	0	0	1	1
siRNA_718189	+	*afl* ep	121	UUGUCAUCUCUACAGCCAUUCCC	23	0	0	0	0	0	2
siRNA_300276	+	*afl* ep	121	UUGUCAUCUCUACAGCCAUUCC	22	0	0	0	0	2	4
siRNA_713703	-	*afl* ep	122	UGUCAUCUCUACAGCCAUUCCCC	23	0	0	0	3	0	0
siRNA_1494944	-	*afl* ep	122	UGUCAUCUCUACAGCCAUUC	20	0	0	0	0	0	1
siRNA_2892684	+	*afl* ep	122	UGUCAUCUCUACAGCCAUUCC	21	0	0	0	0	0	1
siRNA_840759	-	*afl* ep	122	UGUCAUCUCUACAGCCAUUCCC	22	0	0	1	0	0	1
siRNA_584225	-	*afl* ep	122	UGUCAUCUCUACAGCCAUUCCCCA	24	0	0	0	1	1	3
siRNA_285142	-	*afl* ep	122	UGUCAUCUCUACAGCCAUUCC	21	0	0	0	0	4	3
siRNA_3057209	-	*afl* ep	123	GUCAUCUCUACAGCCAUU	18	0	0	1	0	0	0
siRNA_3798662	-	*afl* ep	123	GUCAUCUCUACAGCCAU	17	0	0	0	1	0	0
siRNA_1820983	-	*afl* ep	123	GUCAUCUCUACAGCCAUUCC	20	0	0	0	0	1	0
siRNA_594300	-	*afl* ep	123	GUCAUCUCUACAGCCAUUCCCC	22	0	0	1	0	1	1
siRNA_2047010	+	*afl* ep	123	GUCAUCUCUACAGCCAUUCCC	21	0	0	0	0	0	1
siRNA_436166	-	*afl* ep	123	GUCAUCUCUACAGCCAUUCCC	21	0	0	0	1	1	2
siRNA_2036398	-	*afl* ep	124	UCAUCUCUACAGCCAUUCCCC	21	0	0	0	1	0	0
siRNA_1958686	-	*afl* ep	124	UCAUCUCUACAGCCAUUCCCCA	22	0	0	0	0	1	1
siRNA_2176124	+	*afl* ep	124	UCAUCUCUACAGCCAUUCCC	20	0	0	0	0	0	1
siRNA_2891644	-	*afl* ep	124	UCAUCUCUACAGCCAUUCCCCAGA	24	0	0	0	0	0	1
siRNA_2565266	+	*afl* ep	124	UCAUCUCUACAGCCAUUCCCCA	22	0	0	0	0	0	1
siRNA_3086028	+	*afl* ep	124	UCAUCUCUACAGCCAUUCCCCAGA	24	0	0	0	0	0	1
siRNA_251168	+	*afl* ep	124	UCAUCUCUACAGCCAUUCCCC	21	0	0	0	0	3	6
siRNA_3232950	+	*afl* ep	125	CAUCUCUACAGCCAUUCCCC	20	0	0	0	0	0	1
siRNA_1423799	-	*afl* ep	125	CAUCUCUACAGCCAUUCCCCAGAU	24	0	0	0	0	0	1
siRNA_1296631	-	*afl* ep	125	CAUCUCUACAGCCAUUCCCCA	21	0	0	0	0	1	1

siRNA_2027708	+	*afl* ep	126	AUCUCUACAGCCAUUCCCCAGA	22	0	0	0	0	0	1
siRNA_1215939	-	*afl* ep	126	AUCUCUACAGCCAUUCCCCAG	21	0	0	0	0	0	1
siRNA_2650329	-	*afl* ep	127	UCUCUACAGCCAUUCCCCAGA	21	0	0	0	1	0	0
siRNA_4185026	-	*afl* ep	127	UCUCUACAGCCAUUCCCCAGAUCACGGA	28	0	0	0	1	0	0
siRNA_3313380	+	*afl* ep	127	UCUCUACAGCCAUUCCCCA	19	0	0	0	1	0	0
siRNA_805044	-	*afl* ep	127	UCUCUACAGCCAUUCCCC	18	0	0	1	1	0	0
siRNA_845760	-	*afl* ep	127	UCUCUACAGCCAUUCCCCAGAU	22	0	0	0	0	1	1
siRNA_1818958	-	*afl* ep	127	UCUCUACAGCCAUUCCCCAGAUCA	24	0	0	0	0	0	2
siRNA_838459	+	*afl* ep	127	UCUCUACAGCCAUUCCCCAGA	21	0	0	0	0	0	2
siRNA_2586998	-	*afl* ep	128	CUCUACAGCCAUUCCCCA	18	0	0	0	1	0	0
siRNA_3165063	-	*afl* ep	129	UCUACAGCCAUUCCCCAGAU	20	0	0	0	1	0	0
siRNA_3637026	-	*afl* ep	129	UCUACAGCCAUUCCCCAGAUCACG	24	0	0	1	0	0	0
siRNA_1547223	-	*afl* ep	131	UACAGCCAUUCCCCAGAU	18	0	0	1	1	0	0
siRNA_947778	-	*afl* ep	131	UACAGCCAUUCCCCAGA	17	0	0	0	2	0	0
siRNA_3580499	-	*afl* ep	132	ACAGCCAUUCCCCAGAUC	18	0	0	0	1	0	0
siRNA_4261215	-	*afl* ep	132	ACAGCCAUUCCCCAGA	16	0	0	0	1	0	0
siRNA_1150463	-	*afl* ep	132	ACAGCCAUUCCCCAGAUCACG	21	0	0	0	0	1	0
siRNA_2721454	-	*afl* ep	133	CAGCCAUUCCCCAGAUCACGGACG	24	0	0	0	0	0	1
siRNA_2468100	-	*afl* ep	134	AGCCAUUCCCCAGAUCACG	19	0	0	0	1	0	0
siRNA_2531031	+	*afl* ep	134	AGCCAUUCCCCAGAUCACGGACG	23	0	0	0	0	1	0
siRNA_2135751	-	*afl* ep	134	AGCCAUUCCCCAGAUCACGGACGAAUCCCC	30	0	0	1	0	0	0
siRNA_3412550	+	*afl* ep	134	AGCCAUUCCCCAGAUCACGGACGAA	25	0	0	0	0	0	1
siRNA_380400	-	*afl* ep	134	AGCCAUUCCCCAGAUCACGGA	21	0	0	0	0	1	4
siRNA_4036193	-	*afl* ep	135	GCCAUUCCCCAGAUCACGG	19	0	0	1	1	0	0
siRNA_2082033	-	*afl* ep	135	GCCAUUCCCCAGAUCACG	18	0	0	0	2	0	0
siRNA_3052562	-	*afl* ep	135	GCCAUUCCCCAGAUCACGGACGAA	24	0	0	1	0	0	0
siRNA_710035	-	*afl* ep	135	GCCAUUCCCCAGAUCACGGACG	22	0	0	0	1	2	0
siRNA_1217006	-	*afl* ep	135	GCCAUUCCCCAGAUCACGGA	20	0	0	0	0	1	0
siRNA_2053172	+	*afl* ep	135	GCCAUUCCCCAGAUCACGGACGAAUCCCCUGCAUCU	36	0	0	0	0	0	1
siRNA_789333	+	*afl* ep	135	GCCAUUCCCCAGAUCACGGACGAA	24	0	0	0	0	0	2
siRNA_3626405	-	*afl* ep	136	CCAUUCCCCAGAUCACG	17	0	0	1	0	0	0
siRNA_1592518	+	*afl* ep	136	CCAUUCCCCAGAUCACGGACGAAU	24	0	0	0	0	1	0
siRNA_1275201	+	*afl* ep	137	CAUUCCCCAGAUCACGGACGAAUC	24	0	0	0	0	1	1
siRNA_859994	+	*afl* ep	137	CAUUCCCCAGAUCACGGACGA	21	0	0	0	0	0	2
siRNA_3470799	+	*afl* ep	138	AUUCCCCAGAUCACGGACGAAUCC	24	0	0	0	0	0	1
siRNA_3570496	+	*afl* ep	138	AUUCCCCAGAUCACGGACGAAUC	23	0	0	0	0	0	1
siRNA_3452446	-	*afl* ep	139	UUCCCCAGAUCACGGACGAAUCCCCUGCA	29	0	0	0	1	0	0
siRNA_2869053	-	*afl* ep	139	UUCCCCAGAUCACGGACGAAUCC	23	0	0	0	0	1	0
siRNA_2079702	+	*afl* ep	139	UUCCCCAGAUCACGGACGAAUCCCC	25	0	0	0	1	1	0
siRNA_3488956	-	*afl* ep	139	UUCCCCAGAUCACGGACGAAU	21	0	0	0	0	0	1
siRNA_687997	+	*afl* ep	139	UUCCCCAGAUCACGGACGAAUC	22	0	0	0	0	3	1

siRNA_497221	-	*afl* ep	139	UUCCCCAGAUCACGGACGAAUCCCC	25	0	0	0	2	0	1
siRNA_455199	+	*afl* ep	139	UUCCCCAGAUCACGGACGAAUCCC	24	0	0	0	0	1	4
siRNA_3108343	-	*afl* ep	140	UCCCCAGAUCACGGA	15	0	0	0	1	0	0
siRNA_2179490	-	*afl* ep	140	UCCCCAGAUCACGGACGAAUCCCCUG	26	0	0	0	1	0	0
siRNA_2071116	-	*afl* ep	140	UCCCCAGAUCACGGACGAAUCCCCUGC	27	0	0	1	1	0	0
siRNA_751646	-	*afl* ep	140	UCCCCAGAUCACGGACGAAUCCCCUGCA	28	0	0	1	2	0	0
siRNA_638179	-	*afl* ep	140	UCCCCAGAUCACGGACG	17	0	0	1	2	0	0
siRNA_618831	-	*afl* ep	140	UCCCCAGAUCACGGACGAAUCCC	23	0	0	1	1	1	0
siRNA_485416	-	*afl* ep	140	UCCCCAGAUCACGGACGAAUCC	22	0	0	0	2	1	0
siRNA_4204797	-	*afl* ep	140	UCCCCAGAUCACGGACGAAUCCCCUGCAUC	30	0	0	0	1	0	0
siRNA_667225	-	*afl* ep	140	UCCCCAGAUCACGGACGAAUCCCCU	25	0	0	2	0	0	1
siRNA_106715	-	*afl* ep	140	UCCCCAGAUCACGGACGAAUCCCC	24	0	0	4	12	1	1
siRNA_492842	-	*afl* ep	140	UCCCCAGAUCACGGACGAAUC	21	0	0	1	1	1	1
siRNA_718606	+	*afl* ep	140	UCCCCAGAUCACGGACGAAUCCCC	24	0	0	0	0	1	2
siRNA_660477	+	*afl* ep	140	UCCCCAGAUCACGGACGAAUCC	22	0	0	0	0	1	2
siRNA_447358	+	*afl* ep	140	UCCCCAGAUCACGGACGAAUC	21	0	0	0	0	1	4
siRNA_2230712	-	*afl* ep	141	CCCCAGAUCACGGACGAAUCCCCUGCA	27	0	0	0	1	0	0
siRNA_929123	-	*afl* ep	141	CCCCAGAUCACGGACGAAUCCCC	23	0	0	0	2	0	0
siRNA_1518891	+	*afl* ep	141	CCCCAGAUCACGGACGAAUC	20	0	0	0	0	1	0
siRNA_2148374	-	*afl* ep	141	CCCCAGAUCACGGACGAAUC	20	0	0	0	0	0	1
siRNA_721914	-	*afl* ep	141	CCCCAGAUCACGGACGAAUCCCCU	24	0	0	0	1	0	1
siRNA_464732	+	*afl* ep	141	CCCCAGAUCACGGACGAAUCCCCU	24	0	0	0	0	2	2
siRNA_519157	-	*afl* ep	141	CCCCAGAUCACGGACGAAUCC	21	0	0	0	0	1	3
siRNA_139423	+	*afl* ep	141	CCCCAGAUCACGGACGAAUCC	21	0	0	0	0	5	9
siRNA_111878	+	*afl* ep	141	CCCCAGAUCACGGACGAAUCCCC	23	0	0	0	1	7	9
siRNA_113005	+	*afl* ep	141	CCCCAGAUCACGGACGAAUCCC	22	0	0	0	1	6	11
siRNA_2105557	+	*afl* ep	142	CCCAGAUCACGGACGAAUCCCCU	23	0	0	0	0	1	0
siRNA_1216799	-	*afl* ep	142	CCCAGAUCACGGACGAAUCCCCUG	24	0	0	0	1	0	1
siRNA_1151926	-	*afl* ep	142	CCCAGAUCACGGACGAAUCCC	21	0	0	0	0	1	1
siRNA_481823	-	*afl* ep	142	CCCAGAUCACGGACGAAUCCCC	22	0	0	1	2	0	1
siRNA_899357	+	*afl* ep	142	CCCAGAUCACGGACGAAUCC	20	0	0	0	1	0	2
siRNA_467223	-	*afl* ep	142	CCCAGAUCACGGACGAAUCCCCU	23	0	0	0	0	1	4
siRNA_128820	+	*afl* ep	142	CCCAGAUCACGGACGAAUCCCC	22	0	0	1	1	4	11
siRNA_61031	+	*afl* ep	142	CCCAGAUCACGGACGAAUCCC	21	0	0	1	2	14	18
siRNA_1677834	-	*afl* ep	143	CCAGAUCACGGACGAAUCC	19	0	0	0	0	0	1
siRNA_477960	-	*afl* ep	143	CCAGAUCACGGACGAAUCCCCUG	23	0	0	0	1	2	1
siRNA_435445	-	*afl* ep	143	CCAGAUCACGGACGAAUCCCCUGC	24	0	0	0	1	2	1
siRNA_435358	+	*afl* ep	143	CCAGAUCACGGACGAAUCCCCUGC	24	0	0	0	0	3	2
siRNA_1007440	+	*afl* ep	143	CCAGAUCACGGACGAAUCCCC	21	0	0	0	0	0	2
siRNA_585192	+	*afl* ep	143	CCAGAUCACGGACGAAUCCCCU	22	0	0	0	0	0	3
siRNA_358897	-	*afl* ep	143	CCAGAUCACGGACGAAUCCCCU	22	0	0	0	0	2	4

siRNA_279405	-	*afl* ep	143	CCAGAUCACGGACGAAUCCCC	21	0	0	0	0	3	4
siRNA_3318867	+	*afl* ep	144	CAGAUCACGGACGAAUCCCCUG	22	0	0	0	0	0	1
siRNA_682908	+	*afl* ep	144	CAGAUCACGGACGAAUCCCCUGC	23	0	0	0	0	1	2
siRNA_487799	+	*afl* ep	144	CAGAUCACGGACGAAUCCCCU	21	0	0	0	0	2	2
siRNA_2742687	-	*afl* ep	145	AGAUCACGGACGAAUCCCC	19	0	0	0	1	0	0
siRNA_1676535	-	*afl* ep	145	AGAUCACGGACGAAU	15	0	0	0	1	0	0
siRNA_4113344	-	*afl* ep	145	AGAUCACGGACGAAUCCC	18	0	0	0	0	0	1
siRNA_669374	+	*afl* ep	145	AGAUCACGGACGAAUCCCCUGC	22	0	0	0	0	1	3
siRNA_3573952	+	*afl* ep	146	GAUCACGGACGAAUCCCC	18	0	0	0	1	0	0
siRNA_1297856	-	*afl* ep	146	GAUCACGGACGAAUCCCC	18	0	0	0	1	0	0
siRNA_696220	-	*afl* ep	146	GAUCACGGACGAAUCCCCUGCAUC	24	0	0	0	1	1	0
siRNA_364392	+	*afl* ep	146	GAUCACGGACGAAUCCCCUGC	21	0	0	0	0	2	3
siRNA_752177	+	*afl* ep	147	AUCACGGACGAAUCCCCUGCAUCU	24	0	0	0	0	1	2
siRNA_2042187	+	*afl* ep	148	UCACGGACGAAUCCCCU	17	0	0	0	1	0	1
siRNA_589974	+	*afl* ep	148	UCACGGACGAAUCCCCUGCAUCU	23	0	0	0	0	2	1
siRNA_1573328	+	*afl* ep	148	UCACGGACGAAUCCCCUGCAU	21	0	0	0	0	0	1
siRNA_847245	+	*afl* ep	148	UCACGGACGAAUCCCCUGC	19	0	0	0	0	0	2
siRNA_112565	+	*afl* ep	148	UCACGGACGAAUCCCCUGCAUC	22	0	0	0	0	6	11
siRNA_2530813	-	*afl* ep	149	CACGGACGAAUCCCCUGCAUCU	22	0	0	1	0	0	0
siRNA_1880248	+	*afl* ep	149	CACGGACGAAUCCCCUGCAUCU	22	0	0	0	0	0	1
siRNA_768578	+	*afl* ep	149	CACGGACGAAUCCCCUGCAUC	21	0	0	0	0	2	1
siRNA_2606296	-	*afl* ep	150	ACGGACGAAUCCCCUGCAUCUACG	24	0	0	0	0	0	1
siRNA_3383930	+	*afl* ep	150	ACGGACGAAUCCCCUGCAUCUACG	24	0	0	0	0	0	1
siRNA_718881	+	*afl* ep	150	ACGGACGAAUCCCCUGCAUCU	21	0	0	0	0	0	2
siRNA_1501704	+	*afl* ep	151	CGGACGAAUCCCCUGCAUC	19	0	0	1	0	0	0
siRNA_1685870	+	*afl* ep	151	CGGACGAAUCCCCUGCAUCUAC	22	0	0	0	0	0	1
siRNA_867669	+	*afl* ep	151	CGGACGAAUCCCCUGCAUCUA	21	0	0	0	0	0	2
siRNA_419918	+	*afl* ep	151	CGGACGAAUCCCCUGCAUCUACGC	24	0	0	0	0	1	4
siRNA_2865212	-	*afl* ep	152	GGACGAAUCCCCUGCAUCU	19	0	0	1	0	0	0
siRNA_3449884	-	*afl* ep	152	GGACGAAUCCCCUGCAUCUACGCGCACGC	29	0	0	1	0	0	0
siRNA_1975590	-	*afl* ep	152	GGACGAAUCCCCUGCAUCUACGCGCACGCAU	31	0	0	1	1	0	0
siRNA_851079	-	*afl* ep	152	GGACGAAUCCCCUGCA	16	0	0	0	2	0	0
siRNA_873898	-	*afl* ep	152	GGACGAAUCCCCUGCAUC	18	0	0	0	1	0	0
siRNA_1996583	-	*afl* ep	152	GGACGAAUCCCCUGCAUCUA	20	0	0	0	0	0	1
siRNA_397249	-	*afl* ep	152	GGACGAAUCCCCUGCAUCUACGCG	24	0	0	1	0	2	2
siRNA_590927	-	*afl* ep	152	GGACGAAUCCCCUGCAUCUACGC	23	0	0	0	0	0	4
siRNA_125834	-	*afl* ep	152	GGACGAAUCCCCUGCAUCUAC	21	0	0	0	0	7	8
siRNA_61878	-	*afl* ep	152	GGACGAAUCCCCUGCAUCUACG	22	0	0	1	0	10	23
siRNA_3053404	-	*afl* ep	153	GACGAAUCCCCUGCAUCUACGC	22	0	0	0	1	0	1
siRNA_492032	-	*afl* ep	153	GACGAAUCCCCUGCAUCUACG	21	0	0	0	2	2	1
siRNA_1108219	+	*afl* ep	153	GACGAAUCCCCUGCAUCUACGC	22	0	0	0	0	0	2

siRNA_1494263	-	*afl* J	154	ACGAAUCCCCUGCAUCUACGC	21	0	0	0	0	0	1
siRNA_1330108	+	*afl* J	154	ACGAAUCCCCUGCAUCUACGC	21	0	0	0	0	0	2
siRNA_733352	+	*afl* J	155	CGAAUCCCCUGCAUCUACGCGC	22	0	0	0	0	2	1
siRNA_3234419	+	*afl* J	157	AAUCCCCUGCAUCUACGCGCACGC	24	0	0	0	0	0	1
siRNA_2090072	-	*afl* J	157	AAUCCCCUGCAUCUACGCGCAC	22	0	0	0	0	0	1
siRNA_3604658	-	*afl* J	204	AACAGCAACACAACCUGUCCUCGAUGCGGCUC	32	0	0	1	0	0	0
siRNA_1613138	-	*afl* J	204	AACAGCAACACAACCUGUCCUCGAUGCGGCUCA	33	0	0	1	0	0	0
siRNA_1025590	-	*afl* J	213	ACAACCUGUCCUCGAUGCGGCUC	23	0	0	1	1	0	0
siRNA_1078570	-	*afl* J	213	ACAACCUGUCCUCGAUGCGGCUCA	24	0	0	0	0	1	0
siRNA_471898	+	*afl* J	214	CAACCUGUCCUCGAUGCGGCUC	22	0	0	0	0	0	4
siRNA_2045353	+	*afl* J	216	ACCUGUCCUCGAUGCGGCUCAAUC	24	0	0	0	0	1	0
siRNA_3512880	+	*afl* J	216	ACCUGUCCUCGAUGCGGCUC	20	0	0	0	0	0	1
siRNA_4040742	-	*afl* J	216	ACCUGUCCUCGAUGCGGCUCA	21	0	0	0	0	0	1
siRNA_1326343	+	*afl* J	217	CCUGUCCUCGAUGCGGCUCAA	21	0	0	0	0	1	1
siRNA_3014928	-	*afl* J	219	UGUCCUCGAUGCGGCUCA	18	0	0	0	1	0	0
siRNA_3328580	-	*afl* J	219	UGUCCUCGAUGCGGCUCAAUC	21	0	0	0	1	0	0
siRNA_2029559	+	*afl* J	219	UGUCCUCGAUGCGGCUCAAUC	21	0	0	0	0	0	1
siRNA_1646183	-	*afl* J	220	GUCCUCGAUGCGGCUCAAU	19	0	0	1	1	0	0
siRNA_1762685	-	*afl* J	220	GUCCUCGAUGCGGCUCA	17	0	0	0	1	0	0
siRNA_1065777	-	*afl* J	220	GUCCUCGAUGCGGCUCAAUC	20	0	0	1	0	0	0
siRNA_929941	-	*afl* J	220	GUCCUCGAUGCGGCUCAA	18	0	0	1	1	0	1
siRNA_2344595	+	*afl* J	220	GUCCUCGAUGCGGCUCAAUCAAGG	24	0	0	0	0	0	1
siRNA_1569753	-	*afl* J	220	GUCCUCGAUGCGGCUCAAUCA	21	0	0	0	0	0	1
siRNA_994754	-	*afl* J	220	GUCCUCGAUGCGGCUCAAUCAA	22	0	0	0	0	0	2
siRNA_1224860	-	*afl* J	221	UCCUCGAUGCGGCUCAAU	18	0	0	1	1	0	0
siRNA_570503	-	*afl* J	221	UCCUCGAUGCGGCUCAAUCAA	21	0	0	0	0	1	2
siRNA_2472488	+	*afl* J	222	CCUCGAUGCGGCUCAAUCAAGGC	23	0	0	0	0	1	0
siRNA_2403672	+	*afl* J	222	CCUCGAUGCGGCUCAAUCAAGG	22	0	0	0	0	0	1
siRNA_953324	+	*afl* J	223	CUCGAUGCGGCUCAAUCAAGG	21	0	0	0	0	2	0
siRNA_3463226	+	*afl* J	223	CUCGAUGCGGCUCAAUCAAGGCAACU	26	0	0	0	0	0	1
siRNA_141931	+	*afl* J	223	CUCGAUGCGGCUCAAUCAAGGC	22	0	0	0	0	4	9
siRNA_4178977	+	*afl* J	224	UCGAUGCGGCUCAAUCAAGGCAAC	24	0	0	0	0	0	1
siRNA_306834	+	*afl* J	224	UCGAUGCGGCUCAAUCAAGGC	21	0	0	0	0	2	5
siRNA_3514791	+	*afl* J	225	CGAUGCGGCUCAAUCAAGGCAACU	24	0	0	0	0	1	0
siRNA_1221403	+	*pks* A	227	AUGCGGCUCAAUCAAGGCAACU	22	0	0	0	0	1	1
siRNA_568285	+	*pks* A	227	AUGCGGCUCAAUCAAGGCAACUCU	24	0	0	0	0	1	3
siRNA_2246700	-	*pks* A	228	UGCGGCUCAAUCAAGGCAACUC	22	0	0	0	1	0	0
siRNA_1094892	+	*pks* A	228	UGCGGCUCAAUCAAGGCAACUCUU	24	0	0	0	0	1	0
siRNA_3109786	+	*pks* A	228	UGCGGCUCAAUCAAGGCAACU	21	0	0	0	0	0	1
siRNA_2633760	+	*pks* A	228	UGCGGCUCAAUCAAGGCAACUCU	23	0	0	0	0	0	1
siRNA_4286640	+	*pks* A	228	UGCGGCUCAAUCAAGGCAACUC	22	0	0	0	0	0	1

siRNA_2218969	-	*pks* A	229	GCGGCUCAAUCAAGGCAACUC	21	0	0	1	0	0	0
siRNA_4196304	-	*pks* A	229	GCGGCUCAAUCAAGGCAACUCU	22	0	0	0	1	0	0
siRNA_2447000	+	*pks* A	229	GCGGCUCAAUCAAGGCAACUCU	22	0	0	0	0	0	1
siRNA_1970742	+	*pks* A	229	GCGGCUCAAUCAAGGCAACU	20	0	0	0	0	0	1
siRNA_1516049	+	*pks* A	294	CAGUUUACUAUCCGUCCAGGCCAC	24	0	0	0	0	0	1
siRNA_988669	-	*pks* A	295	AGUUUACUAUCCGUCCAGGCCACG	24	0	0	0	0	1	1
siRNA_3187282	-	*pks* A	296	GUUUACUAUCCGUCCAGGCCACGG	24	0	0	0	0	1	0
siRNA_1393545	-	*pks* A	296	GUUUACUAUCCGUCCAGGCCAC	22	0	0	0	0	0	1
siRNA_3779271	+	*pks* A	297	UUUACUAUCCGUCCAGGCCACG	22	0	0	0	0	0	1
siRNA_1968147	-	*pks* A	299	UACUAUCCGUCCAGGCCACGG	21	0	0	0	1	0	0
siRNA_3270594	+	*pks* A	300	ACUAUCCGUCCAGGCCACGGUAGGGAGGUUUGGGA	38	0	0	0	0	0	1
siRNA_1474562	+	*pks* A	301	CUAUCCGUCCAGGCCACGGUAGGGAGGUUUGGGAC	37	0	0	0	0	0	1
siRNA_1856775	-	*pks* A	302	UAUCCGUCCAGGCCACGGUAG	21	0	0	0	0	0	1
siRNA_554542	-	*pks* A	303	AUCCGUCCAGGCCACGG	17	0	0	0	3	0	0
siRNA_2647824	-	*pks* A	305	CCGUCCAGGCCACGGUAGGGAGGUUU	26	0	0	1	0	0	0
siRNA_3862497	+	*pks* A	306	CGUCCAGGCCACGGUAGGGAGGUUUGGGACUC	32	0	0	0	0	1	0
siRNA_1347615	-	*pks* A	306	CGUCCAGGCCACGGUAGGGAGG	22	0	0	0	1	0	1
siRNA_1091967	-	*pks* A	307	GUCCAGGCCACGGUAGGGAGG	21	0	0	0	1	0	1
siRNA_1151587	-	*pks* A	308	UCCAGGCCACGGUAGGGAGG	20	0	0	1	2	0	0
siRNA_3000545	-	*pes* 1	311	AGGCCACGGUAGGGAGGUUUGGGACUC	27	0	0	0	1	0	0
siRNA_3853660	-	*pes* 1	311	AGGCCACGGUAGGGAGGUUUGGG	23	0	0	0	1	0	0
siRNA_624279	-	*pes* 1	311	AGGCCACGGUAGGGAGGUU	19	0	0	0	3	0	0
siRNA_376256	-	*pes* 1	311	AGGCCACGGUAGGGAGGU	18	0	0	1	5	0	0
siRNA_182477	-	*pes* 1	311	AGGCCACGGUAGGGAGG	17	0	0	1	11	0	0
siRNA_4165447	-	*pes* 1	311	AGGCCACGGUAGGGAGGUUUGGGA	24	0	0	0	0	0	1
siRNA_623772	-	*pes* 1	311	AGGCCACGGUAGGGAGGUUUGG	22	0	0	1	0	1	1
siRNA_210927	-	*pes* 1	311	AGGCCACGGUAGGGAGGUUUG	21	0	0	0	0	4	4
siRNA_3258574	-	*pes* 1	312	GGCCACGGUAGGGAGGUUUGGGA	23	0	0	0	1	0	0
siRNA_1482071	-	*pes* 1	312	GGCCACGGUAGGGAGGUUUGGGACUCUGGAAUUGA	38	0	0	0	1	0	0
siRNA_698358	-	*pes* 1	312	GGCCACGGUAGGGAGGUU	18	0	0	1	2	0	0
siRNA_335459	-	*pes* 1	312	GGCCACGGUAGGGAGGU	17	0	0	2	4	0	0
siRNA_206620	-	*pes* 1	312	GGCCACGGUAGGGAGG	16	0	0	1	8	0	0
siRNA_1198764	-	*pes* 1	312	GGCCACGGUAGGGAGGUUUGGGAC	24	0	0	0	0	1	0
siRNA_2938279	-	*pes* 1	312	GGCCACGGUAGGGAGGUUUGGGACUCUG	28	0	0	0	0	0	1
siRNA_1971270	+	*pes* 1	312	GGCCACGGUAGGGAGGUUUGGGACUC	26	0	0	0	0	0	1
siRNA_556922	-	*pes* 1	312	GGCCACGGUAGGGAGGUUUGG	21	0	0	0	1	1	2
siRNA_1688671	-	*pes* 1	313	GCCACGGUAGGGAGGU	16	0	0	0	1	0	0
siRNA_1594958	-	*pes* 1	313	GCCACGGUAGGGAGGUUUGGGA	22	0	0	0	0	1	0
siRNA_3680616	-	*pes* 1	313	GCCACGGUAGGGAGGUUUGG	20	0	0	0	0	0	1
siRNA_1022536	-	*pes* 1	313	GCCACGGUAGGGAGGUUUGGG	21	0	0	0	0	1	2
siRNA_1870170	+	*pes* 1	314	CCACGGUAGGGAGGUUUGGGACUC	24	0	0	0	0	1	0

siRNA_1142567	+	*pes* 1	314	CCACGGUAGGGAGGUUUGGGA	21	0	0	0	0	0	2
siRNA_2995512	+	*pes* 1	315	CACGGUAGGGAGGUUUGGGACUC	23	0	0	0	0	0	1
siRNA_449706	+	*pes* 1	315	CACGGUAGGGAGGUUUGGGACU	22	0	0	0	0	1	3
siRNA_1062658	+	*pes* 1	316	ACGGUAGGGAGGUUUGGGACUC	22	0	0	0	0	0	2
siRNA_482734	+	*pes* 1	316	ACGGUAGGGAGGUUUGGGACU	21	0	0	0	0	2	2
siRNA_843454	+	*pes* 1	317	CGGUAGGGAGGUUUGGGACUC	21	0	0	0	0	2	0
siRNA_2437555	+	*pes* 1	317	CGGUAGGGAGGUUUGGGACUCU	22	0	0	0	0	0	1
siRNA_2919926	-	*pes* 1	318	GGUAGGGAGGUUUGGGACUCUGGAAU	26	0	0	0	1	0	0
siRNA_973308	-	*pes* 1	318	GGUAGGGAGGUUUGGGACUCUGGAA	25	0	0	0	2	0	0
siRNA_1071498	-	*pes* 1	318	GGUAGGGAGGUUUGGGACUCUGGA	24	0	0	0	0	1	0
siRNA_3928591	-	*pes* 1	319	GUAGGGAGGUUUGGGACUCUGG	22	0	0	0	0	0	1
siRNA_2048538	-	*pes* 1	319	GUAGGGAGGUUUGGGACUCUG	21	0	0	0	0	0	1
siRNA_1483338	+	*pes* 1	319	GUAGGGAGGUUUGGGACUC	19	0	0	0	0	0	1
siRNA_1337584	+	*pes* 1	319	GUAGGGAGGUUUGGGACUCUGG	22	0	0	0	0	1	1
siRNA_4150125	-	*pes* 1	320	UAGGGAGGUUUGGGACUCUGG	21	0	0	0	0	1	0
siRNA_3351115	+	*pes* 1	320	UAGGGAGGUUUGGGACUCUGG	21	0	0	0	0	0	1
siRNA_589303	+	*pes* 1	320	UAGGGAGGUUUGGGACUC	18	0	0	0	0	1	2
siRNA_1383172	-	*pes* 1	321	AGGGAGGUUUGGGACUCUGGAA	22	0	0	0	1	0	1
siRNA_3703620	+	*pes* 1	321	AGGGAGGUUUGGGACUCUGGA	21	0	0	0	0	0	1
siRNA_1359969	-	*pes* 1	321	AGGGAGGUUUGGGACUCUGGA	21	0	0	0	0	0	2
siRNA_848425	-	*pes* 1	322	GGGAGGUUUGGGACUCUGGAA	21	0	0	0	1	0	1
siRNA_2079877	-	*pes* 1	322	GGGAGGUUUGGGACUCUGGAAU	22	0	0	0	1	0	1
siRNA_3681559	+	*pes* 1	323	GGAGGUUUGGGACUCUGGA	19	0	0	0	1	0	0
siRNA_1359058	-	*pes* 1	323	GGAGGUUUGGGACUCUGGAAUUGA	24	0	0	0	0	0	2
siRNA_533227	-	*pes* 1	323	GGAGGUUUGGGACUCUGGAAUU	22	0	0	0	0	0	3
siRNA_310536	-	*pes* 1	323	GGAGGUUUGGGACUCUGGAAU	21	0	0	0	1	3	4
siRNA_781464	-	*pes* 1	324	GAGGUUUGGGACUCUGGAAUUGAC	24	0	0	0	1	0	2
siRNA_948002	-	*pes* 1	324	GAGGUUUGGGACUCUGGAAUU	21	0	0	0	0	0	2
siRNA_663165	-	*pes* 1	324	GAGGUUUGGGACUCUGGAAUUG	22	0	0	0	1	0	2
siRNA_3425726	-	*pes* 1	325	AGGUUUGGGACUCUGGA	17	0	0	1	0	0	0
siRNA_3685506	-	*pes* 1	325	AGGUUUGGGACUCUGGAA	18	0	0	1	0	0	0
siRNA_3450069	-	*pes* 1	325	AGGUUUGGGACUCUGGAAU	19	0	0	0	1	0	0
siRNA_1383120	-	*pes* 1	325	AGGUUUGGGACUCUGGAAUUG	21	0	0	0	0	0	1
siRNA_319475	-	*pes* 1	325	AGGUUUGGGACUCUGGAAUUGA	22	0	0	0	0	1	4
siRNA_310471	-	*pes* 1	325	AGGUUUGGGACUCUGGAAUUGACA	24	0	0	0	0	1	6
siRNA_1855782	+	*pes* 1	326	GGUUUGGGACUCUGGA	16	0	0	0	1	0	0
siRNA_1704203	-	*pes* 1	326	GGUUUGGGACUCUGGAAU	18	0	0	0	1	0	0
siRNA_1023669	-	*pes* 1	326	GGUUUGGGACUCUGGA	16	0	0	0	2	0	0
siRNA_1672557	-	*pes* 1	326	GGUUUGGGACUCUGGAAUUGACA	23	0	0	0	0	0	1
siRNA_1599585	-	*pes* 1	326	GGUUUGGGACUCUGGAAUUGAC	22	0	0	0	0	0	1
siRNA_426033	-	*pes* 1	326	GGUUUGGGACUCUGGAAUUGACAU	24	0	0	0	0	0	4

siRNA_234557	-	*pes* 1	326	GGUUUGGGACUCUGGAAUUGA	21	0	0	0	0	4	5
siRNA_3487413	-	*pes* 1	327	GUUUGGGACUCUGGAAUUGACAU	23	0	0	0	1	0	0
siRNA_2231410	-	*pes* 1	327	GUUUGGGACUCUGGAAU	17	0	0	0	1	0	0
siRNA_3829742	-	*pes* 1	327	GUUUGGGACUCUGGAAUUGACAUUUCGCG	29	0	0	0	0	0	1
siRNA_617621	-	*pes* 1	327	GUUUGGGACUCUGGAAUUGACA	22	0	0	0	0	1	3
siRNA_323176	-	*pes* 1	327	GUUUGGGACUCUGGAAUUGACAUU	24	0	0	0	0	1	4
siRNA_3508281	-	*pes* 1	329	UUGGGACUCUGGAAUUGACAUUUCGCGUA	29	0	0	0	0	1	0
siRNA_4263246	-	*pes* 1	331	GGGACUCUGGAAUUGAC	17	0	0	0	1	0	0
siRNA_1595242	-	*pes* 1	331	GGGACUCUGGAAUUGACAU	19	0	0	0	1	0	0
siRNA_1647057	-	*pes* 1	331	GGGACUCUGGAAUUGACAUU	20	0	0	0	1	0	0
siRNA_650882	-	*pes* 1	331	GGGACUCUGGAAUUGACA	18	0	0	1	3	0	0
siRNA_610751	-	*pes* 1	331	GGGACUCUGGAAUUGACAUUUC	22	0	0	0	0	2	1
siRNA_897205	-	*pes* 1	331	GGGACUCUGGAAUUGACAUUUCGC	24	0	0	0	0	0	2
siRNA_246023	-	*pes* 1	331	GGGACUCUGGAAUUGACAUUU	21	0	0	0	0	3	4
siRNA_1960848	-	*pes* 1	332	GGACUCUGGAAUUGACAUU	19	0	0	0	1	0	0
siRNA_1503960	-	*pes* 1	332	GGACUCUGGAAUUGACAUUUCGCG	24	0	0	0	0	0	1
siRNA_804721	-	*pes* 1	332	GGACUCUGGAAUUGACAUUUC	21	0	0	0	0	1	2
siRNA_549794	-	*pes* 1	332	GGACUCUGGAAUUGACAUUUCG	22	0	0	0	0	2	2
siRNA_3085715	-	*pes* 1	333	GACUCUGGAAUUGACAUUUCGCG	23	0	0	0	1	0	0
siRNA_761158	-	*pes* 1	333	GACUCUGGAAUUGACAUUUCG	21	0	0	0	0	2	1
siRNA_1205088	-	*pes* 1	333	GACUCUGGAAUUGACAUUUCGCGU	24	0	0	0	1	0	1
siRNA_1276905	-	*pes* 1	333	GACUCUGGAAUUGACAUUUCGC	22	0	0	0	0	0	1
siRNA_1159749	-	*pes* 1	334	ACUCUGGAAUUGACAUUUCG	20	0	0	0	0	1	0
siRNA_3186671	-	*pes* 1	334	ACUCUGGAAUUGACAUUUCGCGU	23	0	0	0	0	0	1
siRNA_776319	-	*pes* 1	334	ACUCUGGAAUUGACAUUUCGC	21	0	0	0	0	1	1
siRNA_859058	-	*pes* 1	334	ACUCUGGAAUUGACAUUUCGCG	22	0	0	0	0	0	2
siRNA_143562	-	*pes* 1	334	ACUCUGGAAUUGACAUUUCGCGUA	24	0	0	0	0	4	10
siRNA_4126476	-	*pes* 1	335	CUCUGGAAUUGACAUUU	17	0	0	1	0	0	0
siRNA_3144233	-	*pes* 1	335	CUCUGGAAUUGACAUU	16	0	0	0	1	0	0
siRNA_1168208	-	*pes* 1	335	CUCUGGAAUUGACAUUUCGCGU	22	0	0	0	0	1	1
siRNA_2088145	-	*pes* 1	335	CUCUGGAAUUGACAUUUCGCGUAC	24	0	0	0	0	0	1
siRNA_191130	-	*pes* 1	335	CUCUGGAAUUGACAUUUCGCG	21	0	0	0	0	6	4
siRNA_2947115	-	*pes* 1	336	UCUGGAAUUGACAUUUCGCGUACUG	25	0	0	0	1	0	0
siRNA_1100036	-	*pes* 1	338	UGGAAUUGACAUUUCGCGUAC	21	0	0	1	0	0	1
siRNA_3565846	+	*pes* 1	339	GGAAUUGACAUUUCGCGUACUGU	23	0	0	0	0	0	1
siRNA_1094347	+	*pes* 1	339	GGAAUUGACAUUUCGCGUACUGUU	24	0	0	0	0	0	2
siRNA_429083	-	*pes* 1	339	GGAAUUGACAUUUCGCGUACUG	22	0	0	1	0	1	2
siRNA_3355920	-	*pes* 1	340	GAAUUGACAUUUCGCGUACUGUUGGGUGGUUUAC	36	0	0	0	1	0	0
siRNA_828443	+	*pes* 1	341	AAUUGACAUUUCGCGUACUGUU	22	0	0	0	0	2	1
siRNA_1147743	+	*pes* 1	341	AAUUGACAUUUCGCGUACUGU	21	0	0	0	0	0	2
siRNA_517306	+	*pes* 1	341	AAUUGACAUUUCGCGUACUGUUGG	24	0	0	0	0	1	2

siRNA_3322998	+	*pes* 1	342	AUUGACAUUUCGCGUACUGUUGGG	24	0	0	0	0	0	1
siRNA_650143	+	*pes* 1	342	AUUGACAUUUCGCGUACUGUU	21	0	0	0	0	1	2
siRNA_788773	+	*pes* 1	343	UUGACAUUUCGCGUACUGUUGG	22	0	0	0	0	0	2
siRNA_3227454	+	*pes* 1	344	UGACAUUUCGCGUACUGUUGGGUG	24	0	0	0	0	0	1
siRNA_450838	+	*pes* 1	344	UGACAUUUCGCGUACUGUUGGG	22	0	0	0	0	2	3
siRNA_3265917	-	*pes* 1	345	GACAUUUCGCGUACUGU	17	0	0	0	1	0	0
siRNA_3695643	+	*pes* 1	345	GACAUUUCGCGUACU	15	0	0	0	1	0	0
siRNA_3698604	-	*pes* 1	345	GACAUUUCGCGUACUGUUGGGUGGUU	26	0	0	0	1	0	0
siRNA_3144638	-	*pes* 1	345	GACAUUUCGCGUACUGUUGGGUG	23	0	0	0	0	1	0
siRNA_1316745	+	*pes* 1	345	GACAUUUCGCGUACUGUUGGGUGG	24	0	0	0	0	1	0
siRNA_628818	-	*pes* 1	345	GACAUUUCGCGUACUGUUGGG	21	0	0	1	1	1	1
siRNA_4081234	-	*pes* 1	345	GACAUUUCGCGUACUGUUGG	20	0	0	0	0	0	1
siRNA_462853	-	*pes* 1	345	GACAUUUCGCGUACUGUUGGGU	22	0	0	0	0	2	2
siRNA_1092601	+	*pes* 1	345	GACAUUUCGCGUACUGUUGGGU	22	0	0	0	0	0	2
siRNA_610697	+	*pes* 1	345	GACAUUUCGCGUACUGUUGGG	21	0	0	0	0	1	2
siRNA_341483	-	*pes* 1	345	GACAUUUCGCGUACUGUUGGGUGG	24	0	0	0	0	2	3
siRNA_3957546	-	*pes* 1	346	ACAUUUCGCGUACUGUUGGG	20	0	0	0	1	0	0
siRNA_1507533	+	*pes* 1	346	ACAUUUCGCGUACUGUUGGGUG	22	0	0	0	0	1	0
siRNA_956444	-	*pes* 1	346	ACAUUUCGCGUACUGUUGGGUGGU	24	0	0	0	0	1	1
siRNA_934601	+	*pes* 1	346	ACAUUUCGCGUACUGUUGGGUGGU	24	0	0	0	0	1	1
siRNA_404185	-	*pes* 1	346	ACAUUUCGCGUACUGUUGGGU	21	0	0	0	0	2	2
siRNA_518782	+	*pes* 1	346	ACAUUUCGCGUACUGUUGGGU	21	0	0	0	0	1	3
siRNA_333802	-	*pes* 1	346	ACAUUUCGCGUACUGUUGGGUG	22	0	0	0	0	2	4
siRNA_4049206	-	*pes* 1	347	CAUUUCGCGUACUGUUGGGUG	21	0	0	0	0	0	1
siRNA_3722758	-	*pes* 1	347	CAUUUCGCGUACUGUUGGGUGGUU	24	0	0	0	0	0	1
siRNA_1093344	+	*pes* 1	347	CAUUUCGCGUACUGUUGGGUGG	22	0	0	0	0	1	1
siRNA_1000883	+	*pes* 1	347	CAUUUCGCGUACUGUUGGGUGGUU	24	0	0	0	0	1	1
siRNA_1276871	+	*pes* 1	347	CAUUUCGCGUACUGUUGGGUG	21	0	0	0	0	0	1
siRNA_1347067	+	*pes* 1	348	AUUUCGCGUACUGUUGGGUGGU	22	0	0	0	0	1	1
siRNA_3898379	+	*pes* 1	348	AUUUCGCGUACUGUUGGGUGG	21	0	0	0	0	0	1
siRNA_2009029	+	*pes* 1	348	AUUUCGCGUACUGUUGGGUGGUUU	24	0	0	0	0	0	1
siRNA_3283877	+	*pes* 1	349	UUUCGCGUACUGUUGGGUGGUUU	23	0	0	1	0	0	0
siRNA_3972033	+	*pes* 1	349	UUUCGCGUACUGUUGGGUGGUUUA	24	0	0	0	0	0	1
siRNA_455091	+	*pes* 1	349	UUUCGCGUACUGUUGGGUGGU	21	0	0	0	1	1	3
siRNA_328157	+	*pes* 1	349	UUUCGCGUACUGUUGGGUGGUU	22	0	0	0	0	0	6
siRNA_3775663	+	*pes* 1	350	UUCGCGUACUGUUGGGUGGUUU	22	0	0	0	0	0	1
siRNA_1277909	+	*pes* 1	350	UUCGCGUACUGUUGGGUGGUU	21	0	0	0	0	1	1
siRNA_726782	+	*pes* 1	350	UUCGCGUACUGUUGGGUGGUUUAC	24	0	0	0	0	0	2
siRNA_2065179	+	*pes* 1	351	UCGCGUACUGUUGGGUGGUUUA	22	0	0	0	0	0	1
siRNA_626975	+	*pes* 1	351	UCGCGUACUGUUGGGUGGUUU	21	0	0	0	0	0	3
siRNA_278414	+	*pes* 1	351	UCGCGUACUGUUGGGUGGUUUACC	24	0	0	0	0	3	4

siRNA_3601080	-	*pes* 1	352	CGCGUACUGUUGGGUGGUU	19	0	0	1	0	0	0
siRNA_1725283	-	*pes* 1	352	CGCGUACUGUUGGGUGG	17	0	0	0	2	0	0
siRNA_1424421	-	*pes* 1	352	CGCGUACUGUUGGGUGGUUUA	21	0	0	0	0	0	1
siRNA_2418510	+	*pes* 1	352	CGCGUACUGUUGGGUGGUUUAC	22	0	0	0	0	0	1
siRNA_1957562	-	*pes* 1	353	GCGUACUGUUGGGUGG	16	0	0	0	1	0	0
siRNA_1060315	-	*pes* 1	353	GCGUACUGUUGGGUGGUUUAC	21	0	0	0	0	1	1
siRNA_525027	+	*pes* 1	353	GCGUACUGUUGGGUGGUUUACC	22	0	0	0	0	1	2
siRNA_1919799	+	*pes* 1	354	CGUACUGUUGGGUGGUUUACCAUU	24	0	0	0	0	1	0
siRNA_414981	+	*pes* 1	354	CGUACUGUUGGGUGGUUUACC	21	0	0	0	0	3	2
siRNA_3656327	+	*pes* 1	355	GUACUGUUGGGUGGUUUACCAUU	23	0	0	0	0	0	1
siRNA_2652062	-	*pes* 1	356	UACUGUUGGGUGGUUUACCAUUCUG	25	0	0	1	0	0	0
siRNA_1123081	+	*pes* 1	356	UACUGUUGGGUGGUUUACCAU	21	0	0	0	0	1	1
siRNA_895026	+	*pes* 1	356	UACUGUUGGGUGGUUUACCAUU	22	0	0	0	0	0	2
siRNA_4254317	-	*pes* 1	357	ACUGUUGGGUGGUUUACCAUUCU	23	0	0	0	0	0	1
siRNA_1455909	-	*pes* 1	358	CUGUUGGGUGGUUUACCAUUCUGC	24	0	0	0	0	1	0
siRNA_2990783	+	*pes* 1	358	CUGUUGGGUGGUUUACCAUUCUGC	24	0	0	0	0	0	1
siRNA_2268512	-	*pes* 1	360	GUUGGGUGGUUUACCAUUCUGC	22	0	0	0	0	1	0
siRNA_1222102	-	*pes* 1	364	GGUGGUUUACCAUUCUGCACCCAA	24	0	0	0	0	0	1
siRNA_3861085	-	*pes* 1	365	GUGGUUUACCAUUCUGCACCCA	22	0	0	0	0	0	1
siRNA_716549	+	*pes* 1	365	GUGGUUUACCAUUCUGCACCC	21	0	0	0	0	1	1
siRNA_2681169	+	*pes* 1	366	UGGUUUACCAUUCUGCACCCAAU	23	0	0	0	0	0	1
siRNA_3172012	+	*pes* 1	366	UGGUUUACCAUUCUGCACCCAA	22	0	0	0	0	0	1
siRNA_1664366	+	*pes* 1	366	UGGUUUACCAUUCUGCACCC	20	0	0	0	0	0	1
siRNA_1990944	+	*pes* 1	366	UGGUUUACCAUUCUGCACCCA	21	0	0	0	0	0	1
siRNA_2057444	-	*pes* 1	367	GGUUUACCAUUCUGCACCCA	20	0	0	0	0	1	0
siRNA_2562358	-	*pes* 1	367	GGUUUACCAUUCUGCACCCAAU	22	0	0	0	0	0	1
siRNA_189712	-	*pes* 1	367	GGUUUACCAUUCUGCACCCAA	21	0	0	1	0	2	7
siRNA_3964390	+	*pes* 1	368	GUUUACCAUUCUGCACCCAAUA	22	0	0	0	0	0	1
siRNA_3867923	-	*pes* 1	368	GUUUACCAUUCUGCACCCAAUAAC	24	0	0	0	0	0	1
siRNA_1898671	+	*pes* 1	369	UUUACCAUUCUGCACCCAAUA	21	0	0	0	0	1	0
siRNA_597137	+	*pes* 1	369	UUUACCAUUCUGCACCCAAUAACC	24	0	0	0	0	1	2
siRNA_1067592	+	*pes* 1	370	UUACCAUUCUGCACCCAAUAAC	22	0	0	0	0	1	1
siRNA_1535888	-	*pes* 1	371	UACCAUUCUGCACCCAA	17	0	0	1	0	0	0
siRNA_943267	-	*pes* 1	371	UACCAUUCUGCACCCA	16	0	0	0	2	0	0
siRNA_2287756	+	*pes* 1	371	UACCAUUCUGCACCCAAUAAC	21	0	0	0	0	1	0
siRNA_1768855	-	*pes* 1	371	UACCAUUCUGCACCCAAUAACC	22	0	0	0	0	0	1
siRNA_691118	+	*pes* 1	371	UACCAUUCUGCACCCAAUAACC	22	0	0	0	0	1	1
siRNA_3065370	-	*pes* 1	372	ACCAUUCUGCACCCAA	16	0	0	0	1	0	0
siRNA_1313919	+	*pes* 1	372	ACCAUUCUGCACCCAAUAACC	21	0	0	0	0	1	0
siRNA_1451877	+	*pes* 1	373	CCAUUCUGCACCCAAUAACC	20	0	0	0	1	0	0

All data have been deposited in FigShare: doi: **10.6084/m9.figshare.11771628**. The mapping coordinates are available in the different "**comp_6492_**" files.
